# Structural basis for microtubule recognition by the human kinetochore Ska complex

**DOI:** 10.1038/ncomms3964

**Published:** 2014-01-13

**Authors:** Maria Alba Abad, Bethan Medina, Anna Santamaria, Juan Zou, Carla Plasberg-Hill, Arumugam Madhumalar, Uma Jayachandran, Patrick Marc Redli, Juri Rappsilber, Erich A. Nigg, A. Arockia Jeyaprakash

**Affiliations:** 1 Wellcome Trust Centre for Cell Biology, Institute of Cell Biology, University of Edinburgh, Michael Swann Building, Kings Buildings, Mayfield Road, EH9 3JR Edinburgh, UK; 2 Biozentrum, University of Basel, Klingelbergstrasse 50/70, CH-4056 Basel, Switzerland; 3 National Institute of Immunology, Aruna Asaf Ali Marg, New Delhi 110067, India; 4 Department of Biotechnology, Technische Universität Berlin, 13353 Berlin, Germany; 5 These authors contributed equally to this work

## Abstract

The ability of kinetochores (KTs) to maintain stable attachments to dynamic microtubule structures (‘straight’ during microtubule polymerization and ‘curved’ during microtubule depolymerization) is an essential requirement for accurate chromosome segregation. Here we show that the kinetochore-associated Ska complex interacts with tubulin monomers via the carboxy-terminal winged-helix domain of Ska1, providing the structural basis for the ability to bind both straight and curved microtubule structures. This contrasts with the Ndc80 complex, which binds straight microtubules by recognizing the dimeric interface of tubulin. The Ska1 microtubule-binding domain interacts with tubulins using multiple contact sites that allow the Ska complex to bind microtubules in multiple modes. Disrupting either the flexibility or the tubulin contact sites of the Ska1 microtubule-binding domain perturbs normal mitotic progression, explaining the critical role of the Ska complex in maintaining a firm grip on dynamic microtubules.

## Abstract (web summary)

Kinetochores must interact with both polymerizing (straight) and depolymerizing (curved) microtubules to ensure correct mitotic chromosome segregation. Abad *et al*. reveal how this flexibility is achieved through structural characterization of the interactions between microtubules and the kinetochore protein Ska1.

## Graphical abstract

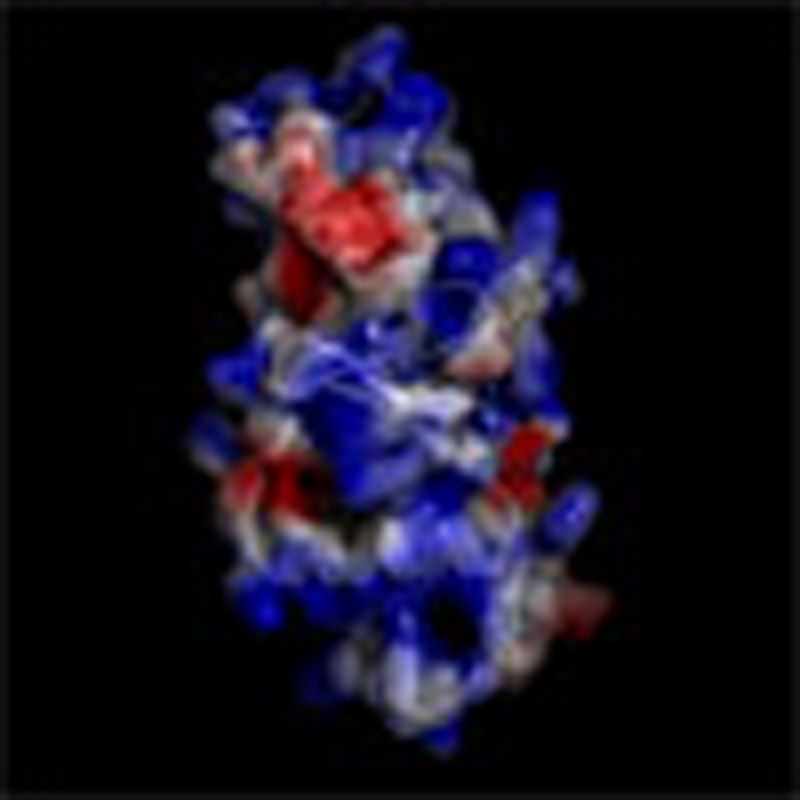

Establishment of physical connections between the chromosomes and the spindle microtubules (MTs) via the kinetochore (KT) is essential for faithfully segregating the duplicated chromosomes to daughter cells^1^,^2^. A key property of the functional KT is its ability to maintain attachments to the plus end of MTs, as they undergo cycles of polymerization and depolymerization commonly known as dynamic instability^3^,^4^,^5^,^6^. As MT depolymerization contributes to the force required for driving chromosome segregation^7^,^8^,^9^,^10^, MT-binding factors that can stay attached to and/or track depolymerizing MTs are essential. At the outer KT, a protein interaction network called the KMN network (consisting of the protein KNL1 and the protein complexes Mis12 and Ndc80) provides the direct binding site for MTs^5^,^11^. Among these components, the Ndc80 complex is the major MT-binding factor, whereas KNL1 also possesses MT-binding ability^11^,^12^.

The Ndc80 complex is a heterotetramer composed of Ndc80, Nuf2, Spc24 and Spc25. The globular domains of Ndc80 and Nuf2 are connected to the globular domains of Spc24 and Spc25 via a long coiled-coil structure, resulting in an ~60-nm dumb-bell-shaped architecture^5^,^13^,^14^. Although the globular heads of Spc24 and Spc25 mediate the KT association, those of Ndc80 and Nuf2 directly interact with MTs^5^,^13^,^15^,^16^. The Ndc80 complex can track depolymerizing MTs when attached to microspheres and it influences MT dynamics by stabilizing straight MTs^17^,^18^. In budding yeast, a ten-subunit protein complex called Dam1 can form a ring around MTs and cooperates with the Ndc80 complex in maintaining stable KT attachments to dynamic MTs^19^,^20^,^21^,^22^. However, no obvious structural Dam1 homologue has been identified in metazoans.

Originally discovered in a proteomics screen^23^, the Ska complex is now recognized as a key element required for maintaining stable KT–MT attachments^24^,^25^,^26^,^27^,^28^,^29^. The ternary Ska complex, composed of Ska1, Ska2 and Ska3, localizes to the outer KT in a KMN-dependent manner. There, it is regulated by the Aurora B kinase^30^, much like the Dam1 complex^19^,^20^. Cells depleted of the Ska complex fail to maintain stable KT–MT attachments, resulting in chromosome congression failure followed by cell death^25^,^26^. The Ska complex can directly interact with MTs and track depolymerizing MTs *in vitro*^26^,^31^. Accordingly, it has been proposed that the Ska and Ndc80 complexes form an integrated MT-binding assembly^31^. The ability of the Ska complex to track depolymerizing MTs *in vitro* and its dependency on the KMN for its localization and function suggest that the Ska complex may be a functional equivalent of the Dam/DASH complex in metazoans^25^,^26^. Besides stabilizing KT–MT attachments, the Ska complex has also been implicated in silencing the spindle checkpoint^24^,^27^,^32^.

During MT growth and shrinkage, MTs undergo important conformational changes. Protofilaments adopt a curved conformation during MT depolymerization and a straight conformation during polymerization. Understanding the structural basis for how the Ndc80 and Ska complexes interact with dynamic MTs is indispensable for understanding the mechanistic aspects of KT–MT attachments. Structural characterizations of Ndc80–MT interactions have shown that the Ndc80 complex binds MTs by interacting at the dimeric interface of α- and β-tubulins. This mode of interaction is also thought to influence the plus-end dynamics of MTs^33^,^34^. Although no atomic structure of the Dam1 complex is available, electron microscopy studies have provided insight into how multimeric Dam1 complexes assemble into a ring-like structure encircling MTs^19^,^35^. Our previous work demonstrated that the Ska complex is a dimer of triple helical bundles formed by Ska1, Ska2 and Ska3, resulting in a W-shaped structure with a maximum interatomic distance of ~350 Å (ref. 36). The MT-binding domains (MTBDs) of the Ska complex protrude at the ends of the W-shaped homodimer, suggesting a transversal mode of MT binding at the KT–MT interface^36^. A recently reported nuclear magnetic resonance (NMR) structure of the *Caenorhabditis elegans* Ska1–MTBD revealed the involvement of a winged-helix domain in MT recognition^31^. At this point, information on how the Ska complex interacts with MTs is crucial to understand the role of the Ska complex in potentially coupling MT dynamics and chromosome segregation. By combining X-ray crystallography, crosslinking/mass spectrometry (MS) and biochemistry, we have here characterized the MTBD of the human Ska complex and evaluated its interaction with MTs *in vitro* and *in vivo*. We show that the Ska complex, unlike the Ndc80 complex, can bind tubulin monomers in different orientations via its multiple MT contact sites, allowing it to recognize MTs in a conformation-independent manner. These results provide novel structural and functional insights into the role of the Ska complex in maintaining stable attachments to dynamic MTs.

## Results

### Ska1_92–255_ is essential for correct mitotic progression

**Figure 1:**
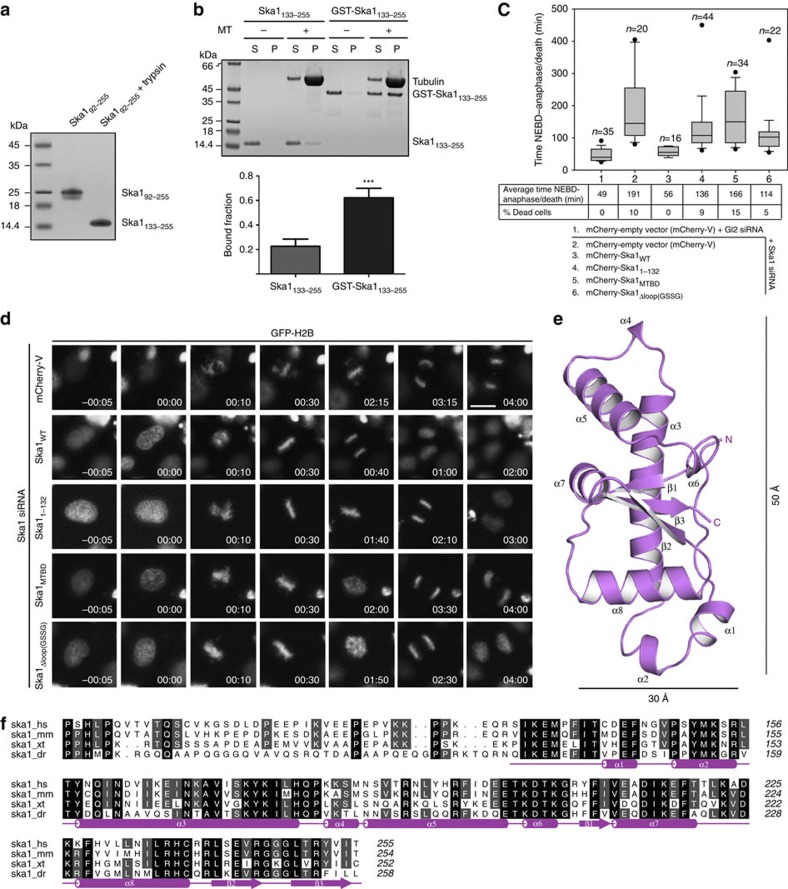
Characterization of functional determinants of Ska1. (**a**) Limited proteolysis of the Ska1_92–255_ with trypsin that led to the formation of a stable fragment identified by MS as Ska1_133–255_. Uncropped scan of the gel is shown in Supplementary Fig. S8a. (**b**) Top, representative SDS–PAGE of cosedimentation assays comparing the MT-binding activity of Ska1_133–255_ and GST–Ska1_133–255_. Bottom, quantification of the MT-binding assays in **b** (mean±s.d., *n*=4, ***P
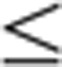
0.001, *t*-test). (**c**) Box-and-whisker plot showing the elapsed time (min) between nuclear envelope breakdown (NEBD) and anaphase onset/death for individual cells. The total number of cells (*n*) from two or more independent experiments is given above each box. Lower and upper whiskers represent 10th and 90th percentiles, respectively. Table summarizing information from the live cell experiments shown below regarding the average time in mitosis (from NEBD until anaphase onset/cell death) and the percentage of cells dying in mitosis. (**d**) Representative stills from time-lapse video-microscopy experiments illustrating mitotic progression of HeLa S3 cells stably expressing histone H2B-GFP treated as in **c**. Time in h:min is indicated. *T*=0 was defined as the time point at which NEBD became evident. Scale bar, 10 μm. (**e**) Cartoon representation of the structure of human Ska1–MTBD, which possesses a modified winged-helix domain with an elongated shape. The length of the structure is ~50 Å whereas the width is ~30 Å. Secondary structure elements are labelled. (**f**) Sequence alignment of human Ska1_91–255_ showing amino acid conservation between *H. sapiens* (hs), *Mus musculus* (mm), *Xenopus tropicalis* (xt) and *Danio rerio* (dr). Secondary structure elements are shown below the aligned sequences. Amino acid conservation is highlighted in grey.

We previously demonstrated that the C-terminal domains of Ska1 (Ska1_92–255_) and Ska3 (Ska3_102–412_) are essential for the function of the Ska complex^36^. Deletion of Ska1_92–255_ completely abolished the MT-binding ability of the complex, supporting its role in recognizing MTs^36^. Secondary structure predictions suggested that Ska1_92–255_ possesses a globular domain preceded by an unstructured region of 40 amino acids. Proteolysis experiments using trypsin and MS analysis identified a stable fragment of Ska1 encompassing residues 133–255 (Ska1_133–255_; Fig. 1a). Consistent with our previous work, Ska1_133–255_ showed weak MT binding on its own^17^ (Fig. 1b). As the full-length (FL) Ska complex is a dimer, we asked whether dimerization of Ska1_133–255_ would increase its affinity for MTs. Exploiting the propensity of glutathione *S*-transferase (GST) to dimerize, we tested the ability of GST-fused Ska1_133–255_ to bind MTs. Although GST–Ska1_133–255_ is not strictly equivalent to the FL Ska1 dimer in the native complex, and thus might bind MTs differently, it clearly interacted with MTs more efficiently than Ska1_133–255_ (Fig. 1b). These observations suggest that Ska1_133–255_ (from now on referred to as MTBD) is the major MT-binding element within the Ska complex, which, on dimerization, can bind MTs more efficiently.

We next evaluated the requirement of the Ska1–MTBD and the loop (Ska1_92–132_, referred to as Ska1 loop) connecting the amino-terminal helical domain (Ska1_1–91_) for correct mitotic progression. As reported earlier^25^,^27^,^31^, Ska1 depletion resulted in a significant increase in mitotic timing (Fig. 1c; see Supplementary Fig. S1a for depletion efficiency). A majority of cells showed metaphase-like appearance (Fig. 1d and Supplementary Movie 1), but roughly a third of the cells showed prolonged prometaphase with chromosome congression defects (Supplementary Movie 2), probably reflecting a more complete depletion of the Ska complex^25^,^30^. FL mCherry-Ska1 efficiently rescued depletion of endogenous Ska1 (Supplementary Movie 3) and cells progressed through mitosis comparably with control (GL2-treated) cells (Fig. 1c). Replacement of endogenous Ska1 by Ska1_1–132_ failed to rescue normal progression through mitosis (Fig. 1c): onset of anaphase was delayed and the frequency of apoptosis increased, but most of the cells showed proper chromosome alignment with timings comparable to cells rescued with Ska1 wild type (WT; Fig. 1d, Supplementary Table S1 and Supplementary Movie 4). Moreover, Ska1_1–132_ localized to KTs but failed to decorate spindle MTs and showed no bundling activity when compared with Ska1 WT (Supplementary Fig. S1b,c). These observations are consistent with a previous report^31^ and show the requirement of the Ska1–MTBD for ensuring stable KT–MT interactions and timely mitotic progression, although this domain seems dispensable for initial chromosome alignment. In contrast, replacement of WT Ska1 by Ska1–MTBD resulted not only in delayed anaphase onset and problems in maintaining a tight metaphase plate (Fig. 1c and Supplementary Movie 5) but also increased the time for initial chromosome alignment (Supplementary Table S1). Considering that Ska1–MTBD lacks both the helical domain required for intermolecular interactions involving Ska2 and Ska3, and the loop that precedes it, these results highlight the importance of the N-terminal helical domain and the Ska1 loop for the complete functionality of the complex (Supplementary Fig. S1). Ska1–MTBD decorated the mitotic spindle but failed to localize to KTs (Supplementary Fig. S1b). The removal of amino acids 1–132 of Ska1 promoted the nuclear localization of the Ska1–MTBD, possibly through a nuclear export signal encompassing amino acids 52–58 of Ska1 (ref. 37). This precluded the assessment of the MT-bundling ability of this construct *in vivo* (Supplementary Fig. S1c).

We next sought to specifically evaluate the functional requirement of the Ska1 loop. Transfection of Ska1Δloop(GSSG), where amino acids 92–132 were replaced by a short linker sequence (GSSG), delayed both chromosome alignment and anaphase onset by twofold (Fig. 1c, Supplementary Table S1 and Supplementary Movie 6). It is to be noted that the Ska1Δloop(GSSG) can bind MTs with comparable efficiency to the WT Ska complex in *in vitro* MT-binding assays (Supplementary Fig. S1d). Interestingly, the bulk of the Ska1Δloop(GSSG) mutant showed no or weak KT localization, suggesting a potential role for the loop in mediating intermolecular interactions required for KT localization (Supplementary Fig. S1b). Together, these results suggest that the flexibility associated with the loop region (and/or intermolecular interactions mediated by it) is required for timely progression through mitosis.

### Ska1–MTBD possesses a modified winged-helix motif

To understand the structural basis for the ability of human Ska complex to bind MTs, we obtained crystals of the Ska1–MTBD in two different crystal forms that diffracted X-rays to about 2 Å (Table 1). The structure was determined by single anomalous dispersion (SAD) experiments using crystals obtained from selenomethionine-incorporated samples. Models from crystal form I (space group C222_1_) and II (space group P3_2_) were refined to 2.1 and 2.3 Å with *R* factors of 19.6 and 22.5, and *R*_free_ factors of 25.1 and 26.8, respectively (Table 1), and superpose well with an overall root mean square deviation of 0.6 Å. Residues 133–142 are stabilized in an extended conformation, followed by eight α-helical segments (α1–α8) and a C-terminal β-hairpin (Fig. 1e,f). The structural analysis showed that the Ska1–MTBD is related to a winged-helix domain, a domain known for its ability to bind DNA and in mediating protein–protein interactions^38^,^39^,^40^,^41^,^42^. The Ska1–MTBD differs from the canonical winged-helix domain by the incorporation of two additional modules (Fig. 1e and Supplementary Fig. S2a).

### Human and *C. elegans* Ska1–MTBDs show structural variations

During the course of this work, the NMR structure of the MTBD of the *C. elegans* Ska1 was reported^31^. The Ska1–MTBD of *C. elegans* shares 28% sequence identity and 46% sequence similarity with its human counterpart. Structural comparisons show that the overall topology of the human and *C. elegans* Ska1–MTBDs is the same (structures superpose with an overall root mean square deviation of 3.0 Å). However, in the *C. elegans* structure, helices α5 and α8, and the β-strands β2 and β3 are in a different orientation relative to the rest of the structure, resulting in noticeable changes in the surface charge distribution of the MTBD (Supplementary Fig. S2b–d). Structure-based sequence alignment reveals that *C. elegans* amino acid Thr168 acts as a hinge residue between α3 and α5 (Supplementary Fig. S2c). In contrast, the corresponding amino acid in higher vertebrates is proline (182, human numbering), an amino acid that has limited backbone conformational flexibility (Supplementary Fig. S2c).

To uncover potential differences in the properties of human versus *C. elegans* Ska1–MTBDs, as manifested by sequence and structural variations, 50 ns molecular dynamic simulations (MDS) were carried out using the two structures. The analysis of the root mean squared fluctuations of the Cα atoms during MDS shows that the MTBD domain of *C. elegans* Ska1 has more intrinsic structural flexibility (particularly regions that show conformational variation) than its human counterpart (Supplementary Fig. S2e). Considering the modest sequence similarity between the human and *C. elegans* Ska1–MTBDs, the suggested conformational variability in the respective structures seems reasonable. However, it is to be noted that the structures we are comparing were obtained by crystallography and NMR, respectively; thus, definitive conclusions on the proposed structural variations require further validation.

### Ska interacts with MTs using a multipartite mode of binding

**Figure 2:**
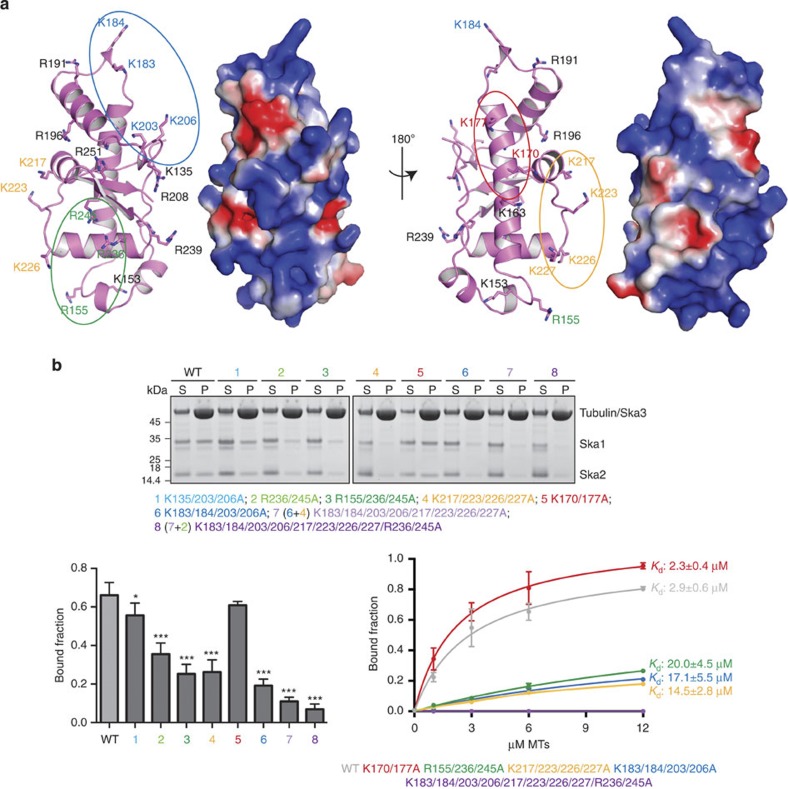
The Ska complex binds MTs through multiple positively charged clusters. (**a**) Cartoon representation of the Ska1–MTBD where surface-exposed K/R residues are shown as sticks (left). Surface representation of the Ska1–MTBD in the same orientation with electrostatic surface potential revealing the presence of positively charged patches (right). Residues clustered based on their proximity and mutated to A to test their involvement in MT recognition are highlighted in different colours. (**b**) Cosedimentation assays of the different K/R- to A-untagged Ska mutants were performed. Representative gels (upper panel) and quantifications of MT cosedimentation assays (bottom panel). Concentration of Ska1 mutants and MTs used in the assays are 3 μM and 6 μM, respectively (mean±s.d., *n*
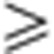
3, *P
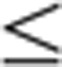
0.05, ***P
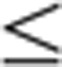
0.001; *t*-test; right bottom panel). *K*_d_ values were calculated using 1 μM Ska and 0–12 μM MTs (bottom left panel). Uncropped scans of the gels are shown in Supplementary Fig. S8b.

The basic nature of the Ska1–MTBD (predicted pI=9.2) led us to hypothesize that the Ska complex recognizes MTs through electrostatic interactions. Analysis of the electrostatic surface potential revealed the existence of contiguous positively charged patches all over the Ska1–MTBD surface (Fig. 2a), suggesting the potential involvement of multiple MT contact sites. Of the 23 Lys (K)/Arg (R) residues that are present in the Ska1–MTBD, 14 are clearly exposed to solvent (Fig. 2a). To identify the critical residues required for MT binding, K/R residues that cluster on the surface were mutated to Ala (A) in the context of the FL human Ska complex. Before subjecting the mutants to MT cosedimentation assays, we analysed their size-exclusion chromatographic profiles to rule out the influence of the mutations on the overall structure of the complex. All the mutants behaved identically to the WT Ska complex, suggesting that mutations do not affect the proper folding of the Ska complex (Supplementary Fig. S3c). Although mutations at K170/177 and K135/203/206 showed no major effects on MT binding, mutations at R155/236/245, a region reported recently to be critical for MT binding^31^, together with two new regions identified in this study, K183/184/203/206 and K217/223/226/227, all showed significant reductions in MT binding (*K*_d_=20±4.5, 17.1±5.5 and 14.5±2.8 μM, respectively, versus WT=2.9±0.6 μM), pointing to the existence of multiple MT interaction sites (Fig. 2b and Supplementary Fig. S3a). Confirming this notion, a combination of R236/245A with R155A resulted in stronger reduction in MT binding than R236/245A alone (Fig. 2b and Supplementary Fig. S3b), and the simultaneous mutation of multiple clusters (K183/184/203/206/217/223/226/227/R236/245A) almost completely abolished MT binding (Fig. 2b). These results provide clear evidence that the human Ska complex binds MTs through a multipartite binding mode of Ska1–MTBD.

### Multipartite MT binding is required for Ska complex function

**Figure 3:**
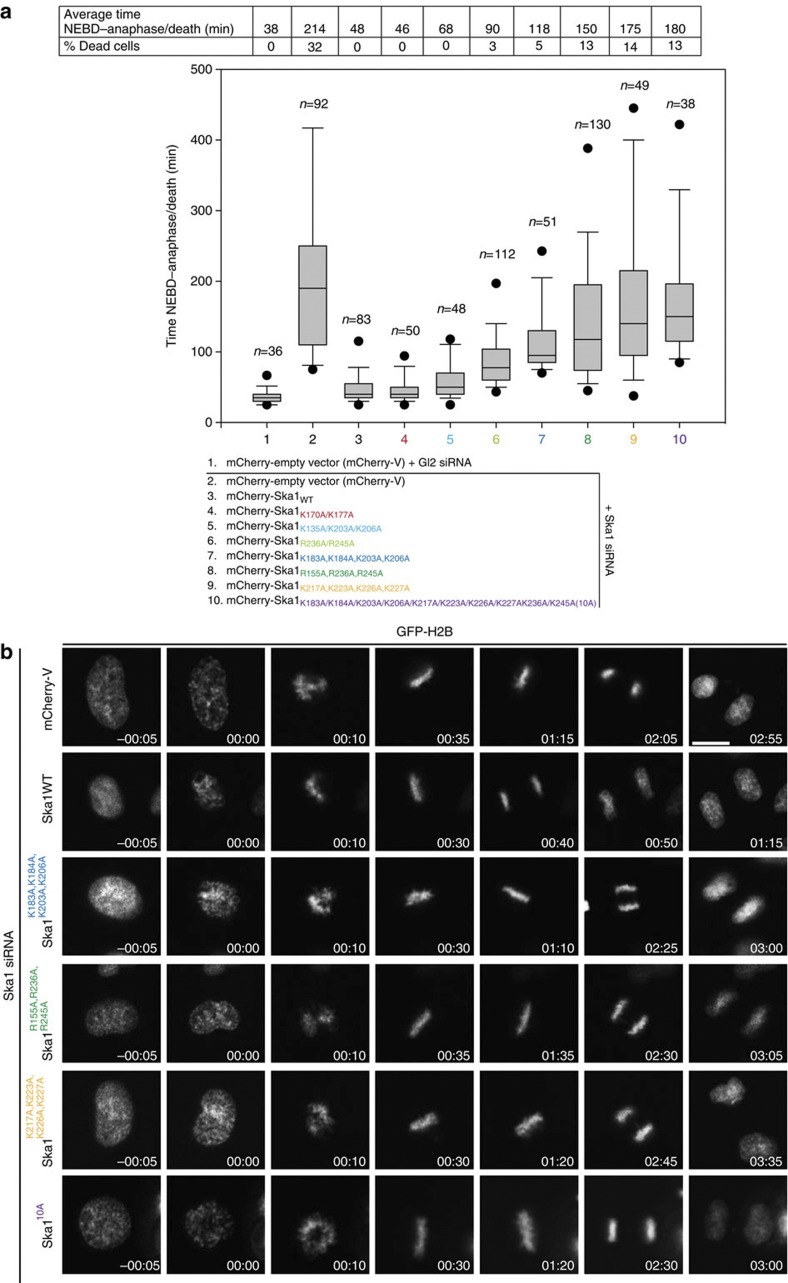
MT recognition of Ska1 via its multipartite mode of MT binding is a functional requirement for the Ska complex. (**a**) Box-and-whisker plot showing the elapsed time (min) between nuclear envelope breakdown (NEBD) and anaphase onset/death for individual cells. The total number of cells (*n*) from two or more independent experiments is given above each box. Lower and upper whiskers represent 10th and 90th percentiles, respectively. Table summarizing information from the live cell experiments shown below regarding the average time in mitosis (from NEBD until anaphase onset/cell death) and the percentage of cells dying in mitosis. (**b**) Representative stills from time-lapse video-microscopy experiments illustrating mitotic progression of HeLa S3 cells stably expressing histone H2B-GFP. Scale bar, 10 μm.

We next evaluated the functional significance of the positively charged clusters of the Ska1–MTBD using small interfering RNA (siRNA) rescue assays with the above K or R to A mutants. In line with *in vitro* results, cells transfected with K170/177A and K135/203/206A mutants showed normal mitotic progression (Fig. 3). Similar to WT cells, cells transfected with K170/177A kept the ability to strongly bundle MTs (Supplementary Fig. S4a), highlighting the preserved MT-binding activity in this mutant. Transfection of the Ska1 K135/203/206A mutant resulted in fewer and weaker MT bundles in interphase cells (Supplementary Fig. S4a), most likely to be reflecting a role of K203/206 in MT binding (Fig. 2b); as shown below, this becomes apparent in combination with mutations at other residues (see below). In contrast, rescues by the Ska1 K183/184/203/206A, R155/236/245A and K217/223/226/227A mutants resulted in perturbed mitotic progression, characterized by a prolonged delay in anaphase onset and an increase in the number of apoptotic cells, but no MT bundling in interphase cells (Fig. 3, Supplementary Fig. S4a and Supplementary Table S2). In agreement with the results of the *in vitro* MT-binding assay, the R236/245A mutant on its own showed a milder phenotype, but when combined with R155A it showed a much more pronounced phenotype (Fig. 3), suggesting cooperation between R236/245A and R155A.

All Ska1 mutants tested were able to form a complex with Ska2 and Ska3 (Fig. 2b), and they localized to KTs, indistinguishable from WT Ska1 (Supplementary Fig. S4b), indicating that the mitotic defects described above are due to interference with MT binding. Indeed, the majority of cells expressing R155/236/245A, K217/223/226/227A and K183/184/203/206A contained aligned chromosomes (Fig. 3b, Supplementary Table S2 and Supplementary Movies 7–9, respectively), reminiscent of Ska1–MTBD expression (see above) and indicative of the requirement of the MT-binding activity for robust KT–MT attachments, but not for the initial contact between KTs and MTs.

### Ska1 interacts with tubulin monomers at multiple sites

Having established a multipartite mode of MT binding by the Ska complex, we next aimed at identifying the structural features of MTs that are recognized by the Ska complex. For this purpose, we crosslinked the Ska1–MTBD/Ska complex with MTs, using 1-ethyl-3-[3-dimethylaminopropyl] carbodiimide hydrochloride (EDC). This reagent crosslinks K (and less favourably S, T, Y) to E or D. Analysis of the crosslinked products in SDS–polyacrylamide gel electrophoresis (SDS–PAGE) showed a predominant band that migrated at the expected molecular weight for one Ska1–MTBD/Ska1 crosslinked to an α-/β-tubulin monomer (marked by asterisks in Supplementary Fig. S5a,b).

**Figure 4:**
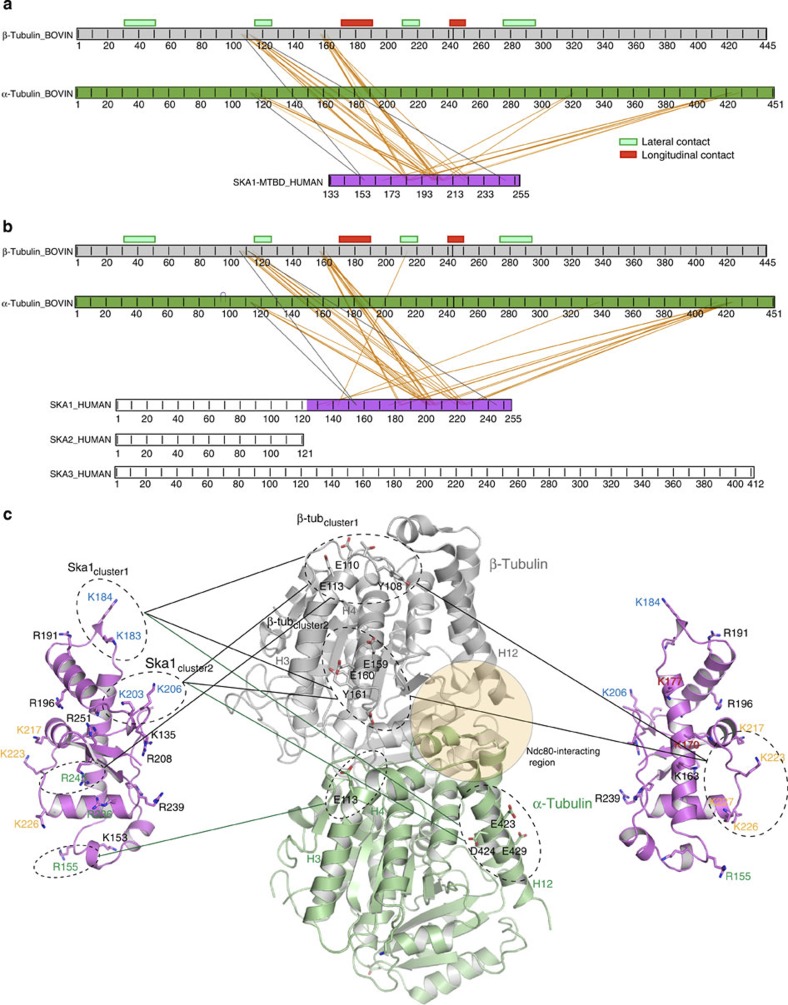
Ska1 interacts with MTs by recognizing the globular regions of tubulin monomers in multiple orientations. (**a**,**b**) Linkage map showing the sequence position of all the crosslinked residue pairs between (**a**) Ska1–MTBD (**b**) Ska complex and α1B and β2B tubulin, where 6 μM human Ska complex/Ska1–MTBD was incubated with 10 μM of MTs in the crosslinking reactions. Crosslinked products were resolved in SDS–PAGE followed by MS analysis. Red and green boxes above the β-tubulin show regions of tubulin involved in longitudinal and lateral contacts, respectively. Crosslinks observed between Ska1 R155/236/245K mutant and MTs are shown in grey. (**c**) Cartoon representation of tubulin dimer where residues involved in crosslinking with Ska1 residues are highlighted in stick representation. Grey and green lines denote crosslinks observed between K/R clusters of Ska1 and Glu/Asp/Tyr/Thr clusters of β-tubulin and α–tubulin, respectively. The region where Ndc80 interacts with tubulin as reported in Alushin *et al*.^33^ is shown in yellow. Important Ska1 residues involved in MT binding are colour coded as in Figs 2 and 3.

MS analysis of the crosslinked products allowed us to pinpoint the residues involved in intermolecular recognition between the Ska complex and tubulins (Fig. 4 and Supplementary Fig. S5e). The overall sequence coverage for Ska1/Ska1–MTBD and tubulin monomers was almost complete, except for the flexible C-terminal tails of tubulin (Supplementary Fig. S5f). Consistent with our biochemical and functional analyses, most of the crosslinks observed for the FL Ska complex bound to MTs involved the MTBD of Ska1. The Ska1–MTBD made almost identical crosslinks with MTs, regardless of whether it was analysed on its own or in the context of the Ska complex, highlighting the specificity of the interaction (Fig. 4a,b). Furthermore, Ska2, which has been shown not to have any MT-binding activity, did not produce any crosslinked peptides with tubulin, confirming the specificity of the crosslinking reaction. Among the three K/R clusters that we identified to be crucial for MT binding and function, two (K183/184/203/206 and K216/217/223/226) showed crosslinks with tubulin monomers. Mapping of the crosslinked residues on the three-dimensional structures of Ska1–MTBD and MTs showed that these clusters contact globular/folded regions of tubulin monomers (unlike most MT-binding proteins that interact with MTs by recognizing acidic tails of tubulins) mainly at two helices: H3 and H4 of β-tubulin and H3 and H12 of α-tubulin (Fig. 4c). Interestingly, intermolecular contacts of Ska1 with H4 of β-tubulin and H12 of α-tubulin seem to be sequence specific, as these tubulin residues are unique to α- and β-isoforms (results not shown).

To rule out the possibility that the crosslinking peptides observed are due to nonspecific interactions with free tubulin monomers, we pelleted MTs crosslinked to Ska1–MTBD before MS analysis and compared the results with those obtained from non-pelleted samples (Fig. 4). Analysis of both samples by SDS–PAGE showed identical crosslinked products (Supplementary Fig. S5g). Furthermore, the contact sites observed were almost identical in both pelleted and non-pelleted samples (Supplementary Fig. S5h), attesting to the specificity of the interactions.

In this analysis, we did not detect crosslinks involving the R155/236/245 cluster. This, as well as the fact that we did not detect acidic tails of tubulin monomers, may be due to the following technical reasons: first, EDC, the crosslinking reagent used in this study, does not crosslink arginines; second, peptides derived from the acidic tails of tubulins may have escaped detection by MS possibly because of the presence of posttranslational modifications (notably polyglutamylation) and/or the lack of tryptic cleavage sites, which would result in large peptide fragments that cannot be detected in crosslinked/MS analysis. To overcome the former limitation, we have mutated R155/236/245 to lysine residues in the context of both Ska1–MTBD and FL Ska1, and then tested these mutants in crosslinking/MS experiments (Supplementary Fig. S5c,d). Indeed, the R155/236/245K mutant did reveal interactions between K155 and K245 of Ska1 with H3 of α- and β-tubulin, respectively (Fig. 4c).

### Ska complex can bind MTs in multiple different orientations

The Lys clusters of Ska1 and the Asp/Glu clusters of tubulin monomers crosslinked in different ways with each other, suggesting the presence of multiple modes of Ska–MT interactions (Fig. 4c). For example, the K183/184 cluster (Ska1_cluster1_) crosslinked with both E110/E113/Y108 (β-tub_cluster1_) and E159/E160/Y161 (β-tub_cluster2_) clusters of β-tubulin and the same could also be observed for the K203/206 cluster (Ska1_cluster2_; Fig. 4c). Moreover, the distance between Ska1_cluster1_ and Ska1_cluster2_ is the same as that between β-tub_cluster1_ and β-tub_cluster2_. Straightforward rigid body docking experiments show that Ska1 can interact with β-tubulin with Ska1_cluster1_–Ska1_cluster2_, either facing β-tub_cluster1_–β-tub_cluster2_ or β-tub_cluster2_–β-tub_cluster1._ In addition, each Ska1 Lys cluster can interact individually with all tubulin Asp/Glu clusters. However, the assertion on the ability of the Ska complex to interact with MTs in multiple different orientations needs further validation.

### Ska and Ndc80 complexes recognize different features of MTs

**Figure 5:**
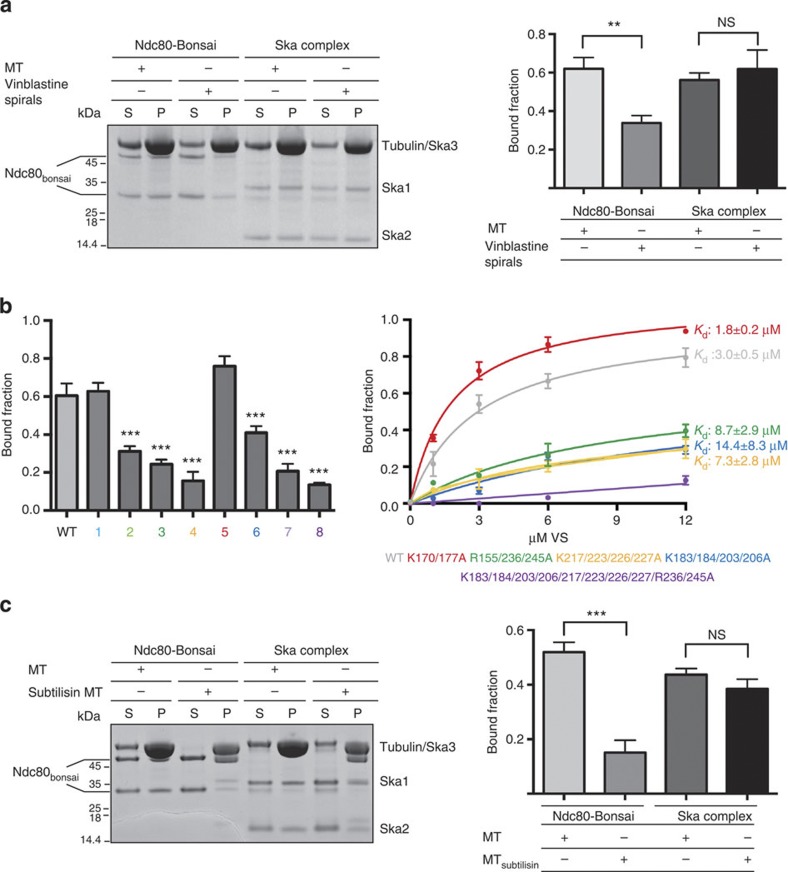
Ska and Ndc80 complexes recognize different structural features of MTs. (**a**) Left, representative SDS–PAGE. Right, quantification of MT cosedimentation assays with Ndc80 and Ska complexes (1 μM) binding to taxol-stabilized MTs or curved protofilaments induced by vinblastine (6 μM). (**b**) Quantifications of MT cosedimentation assays with 3 μM Ska and 6 μM vinblastine spirals (right panel) (mean±s.d., *n*
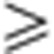
3, ***P
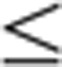
0.001; *t*-test). *K*_d_ values were calculated using 1 μM Ska and 0–12 μM vinblastine spirals (left panel). (**c**) Left, representative SDS–PAGE. Right, quantification of MT cosedimentation assays with Ndc80 and Ska complexes (1 μM) binding to MTs or subtilisin-treated MTs (6 μM; mean±s.d., *n*
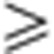
3, ***P
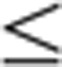
0.001; *t*-test). Uncropped scans of the gels are shown in Supplementary Fig. S8c.

Structural characterizations of Ndc80–MT interactions revealed that the Ndc80 complex binds MTs by recognizing the dimeric interface of α- and β-tubulins^33^. This mode of MT binding by the Ndc80 complex makes the interaction sensitive to the conformation of MT protofilaments. Indeed, the Ndc80 complex preferentially binds straight MTs over curved MT protofilaments (vinblastine spirals; Fig. 5a and Supplementary Fig. S6b), in line with previous reports^31^,^33^. Our crosslinking/MS and cosedimentation data presented here show that the Ska complex, in contrast to the Ndc80 complex, interacts with MTs by recognizing the regions of tubulin monomers whose accessibility is not perturbed when MTs adopt different conformations. Thus, the Ska complex can bind straight and curved MT protofilaments indiscriminately using the same contact sites (see *K*_d_ values for straight MTs in Fig. 2b and corresponding values for vinblastine spirals in Fig. 5b). In future, it will be interesting to explore the possibility that the presence of the Ndc80 complex (or other Ska-binding partners) could induce some of the binding sites to discriminate between different MT structures.

MT-interacting proteins often interact with MTs by recognizing the acidic tails of tubulin, called ‘E-hooks’. Biochemical and structural characterizations of Ndc80–MT interactions carried out in different laboratories have also highlighted the important contribution of the acidic tail of tubulin in the overall recognition of MTs by the Ndc80 complex^13^,^33^. Our crosslinking MS analysis does not provide insight into the possible role of acidic tubulin tails in Ska complex binding, owing to the technical reasons discussed in the previous section. However, as the Ska complex makes multiple contacts with the structured regions of tubulins, we hypothesized that the acidic tail of tubulin may not significantly contribute to the overall recognition of MTs. To evaluate this hypothesis, we tested the ability of the Ska complex to bind subtilisin-treated MTs (where E-hooks are removed by subtilisin treatment) in cosedimentation assays. As expected, the Ndc80 complex showed reduced binding to subtilisin MTs. Interestingly, the Ska complex did not show any noticeable reduction in its ability to bind subtilisin MTs, in line with the view that the critical contacts involve the structured regions of tubulin monomers rather than the acidic tails (Fig. 5c and Supplementary Fig. S6c).

### Aurora B sites lie within the MT-binding K/R clusters

**Figure 6:**
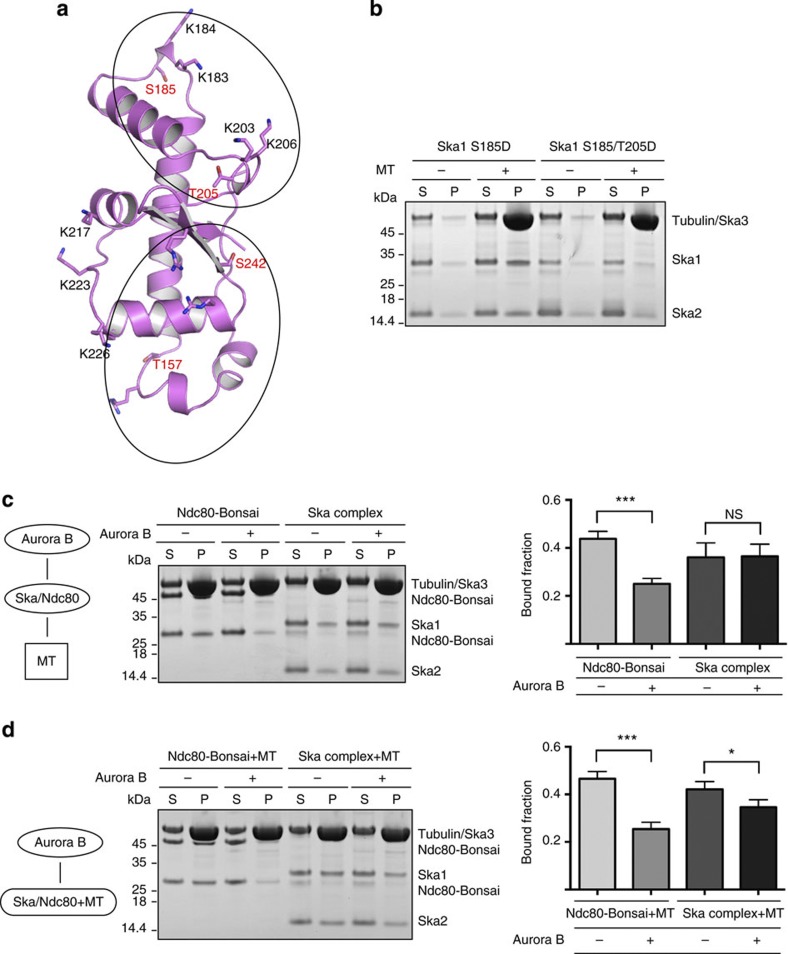
Constitutive phosphorylation of S185/T205 perturbs MT binding of the Ska1–MTBD *in vitro*. (**a**) Cartoon representation of Ska1–MTBD, where consensus Aurora B phosphorylation sites are shown in stick representation (red). The phosphorylation sites of Aurora B are located within two of the three main clusters responsible for the MT-binding activity of Ska1 (circled). (**b**) Representative SDS–PAGE of the MT cosedimentation assays where phosphomimic mutants S185D and S185/T205D are tested. (**c**,**d**) Left, schematic representation of both protocols used for the treatment of Ska or Ndc80 complex with Aurora B. Middle, representative SDS–PAGE of the cosedimentation assays, where (**c**) the Ska/Ndc80 complex was incubated first with Aurora B kinase and subsequently with MTs. (**d**) MT-bound Ska/Ndc80 complex was incubated with Aurora B. Right, quantification of the results obtained (mean±s.d., *n*
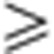
3, *P
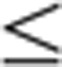
0.05, ***P
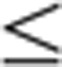
0.001; *t*-test). Uncropped scans of the gels are shown in Supplementary Fig. S8d.

The role of Aurora B kinase in correcting erroneous KT–MT attachments by phosphorylating components of the KMN network, notably the Ndc80 complex and KNL1, is well established^11^,^34^,^43^,^44^. Recent work also showed that Aurora B negatively regulates the KT localization of the Ska complex, possibly influencing interactions with the KMN network^30^. As noted elsewhere^31^, all four Aurora B consensus sites (T157, S185, T205 and S242) are located within the Ska1–MTBD (Fig. 6a). Interestingly, two of these sites (S185 and T205) are located within the K/R cluster K183/184/203/206 that we have identified as being important for the MT-binding activity of the Ska complex (Fig. 6a). This suggested a direct involvement of S185 and T205 in the Aurora B-mediated phosphoregulation of Ska–MT interactions. To test this possibility, we made phosphomimicking mutants of Ska1 (S185D and S185/T205D) and tested them in MT-binding assays. Although S185D did not show any noticeable reduction in its ability to bind MTs, S185/T205D showed a drastic reduction (Fig. 6b and Supplementary Fig. S7). However, in line with our previous study^30^, pre-incubation of the Ska complex with Aurora B did not reduce its MT-binding ability, although the Ndc80 complex analysed for control responded as expected (Fig. 6c). To explain these apparently contradictory findings, we considered the possibility that efficient phosphorylation of Ska1 by Aurora B might require prior conformational rearrangements within the MTBD, which might occur either on MT binding or in the context of other KT-associated proteins. To test the former possibility, we first allowed the Ska complex to bind MTs before incubating the MT-bound Ska complex with Aurora B and evaluating the consequences in MT-binding assays. This experiment revealed a small but statistically significant reduction in the Ska–MT interaction in response to Aurora B (Fig. 6d). In line with the above model, normal mode analysis of the MD simulated structures of Ska1–MTBD shows the presence of an intrinsic flexibility associated with the structural element that harbours this MT-binding site and Aurora B consensus sites (Supplementary Movie 10). The other Aurora B sites, S157 and S242, are also close to the R cluster R155/236/245, but insertion of phosphomimicking mutations did not show any influence on MT binding (Supplementary Fig. S7). It is interesting that combination of a phosphomimic mutation at S242D did not abolish MT binding in combination with T157D (this study), although it has been previously shown that combined mutations at S242D and S185D strongly reduced MT binding. This observation, together with our observation that the S185D/T205D combination also drastically diminishes MT binding, demonstrates that Aurora B phosphorylation of Ska1–MTBD can negatively regulate Ska–MT interactions via multiple phosphorylation events.

## Discussion

MT-binding activity at the KT is a prime requirement for driving accurate chromosome segregation. Although the Ndc80 complex is considered to be the major contributor to MT binding by the KT, other factors such as the Dam1 complex in budding yeast and the Ska complex in vertebrates are required to efficiently couple MT binding at the KT with chromosome segregation^5^. The ability of the Dam1 complex to form rings around MTs suggested that Dam1-like proteins might work as force couplers that harness the force associated with MT depolymerization with chromosome movement^5^,^45^. However, the proposed functional homologue of Dam1 in humans, the Ska complex, forms a flexible W-shaped structure of triple helical bundles with a maximum interatomic distance of 35 nm (ref. 36). With MTBDs symmetrically positioned at both ends of the dimer, the Ska complex thus appears to exert its function via a different mechanism.

Here we have used biochemical and high-resolution structural analysis to show that the human Ska complex interacts with MTs through the C-terminal domain of Ska1, which forms a variant form of winged-helix domain. This domain has previously been seen as a DNA-binding module in transcription regulators but also as a protein interaction module in a small number of proteins with diverse functions^38^,^41^,^42^. The Ska complex provides the first instance where this module is used as a MTBD. However, considering that, first, both DNA molecules and MTs are often recognized through electrostatic interactions exploiting the negatively charged nature of these molecules, and, second, that MT-based diffusional motility and DNA-based diffusion shows striking similarities with comparable diffusion coefficients^46^, the use of a winged-helix domain for the Ska1–MTBD may not be surprising. It will be interesting to see whether the Dam1 complex also possesses winged-helix domains in its MT-binding components.

Structural comparisons of human and *C. elegans* Ska1–MTBDs^31^ show conformational variations in the regions that we demonstrate here to be critical for MT binding. This adds to species-specific differences in the overall composition and architecture of the Ska complexes, in that Ska1 and Ska3 associate in a 2:1 complex in *C. elegans*^31^, whereas the human Ska complex is made of Ska1, Ska2 and Ska3 in a 2:2:2 ratio^36^. It is tempting to suggest that the attachment of chromosomes to spindle MTs in vertebrates versus nematodes may exhibit different dynamic properties. In organisms with ‘holocentric’ chromosomes, such as *C. elegans*, MTs are attached at multiple sites along the chromosome arms, resulting in chromosomes with no apparent dynamic oscillations at metaphase. In contrast, in ‘monocentric’ mammalian cells, chromosomes are attached to MTs at a discrete site and metaphase-aligned chromosomes show pronounced dynamics^47^.

Using MT-binding assays in combination with siRNA-based rescue assays, we demonstrate here the involvement of at least three tubulin contact sites within Ska1–MTBD for MT recognition. Remarkably, the tubulin contact sites are dispersed across the surface of the Ska1–MTBD and distances between different contact sites range between 15 and 30 Å. Disruption of even one of the tubulin contact sites is enough to perturb normal mitotic progression, suggesting the requirement of all contact sites for efficient function. Crosslinking/MS analysis revealed important molecular details of these Ska–MT interactions, in particular the novel ability of the Ska–MTBD to bind to tubulin monomers. This data together with those from quantitative MT cosedimentation assays demonstrated that tubulin contacts of the Ska1–MTBD can bind straight and curved MTs with no apparent preference.

**Figure 7:**
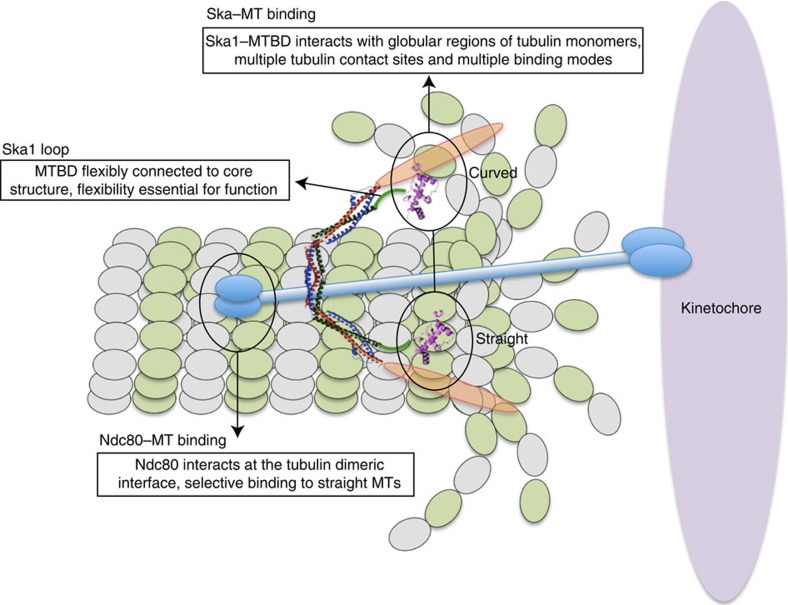
Schematic model. Schematic model summarizing the mode of MT binding of Ska1 and its implications for maintaining stable KT–MT attachments.

Identifying and characterizing the unique properties of the Ndc80 and Ska complexes is crucial for understanding how these complexes complement each other in providing an integrated interface for efficient MT binding and MT-driven motility. The Ndc80 complex binds MTs through the interaction of the Ndc80-CH (Calponin Homology) domain (called the ‘toe’) at the tubulin dimeric interface (called the ‘toe print’) in a way that favours interactions with the straight conformation of MT protofilaments^33^. Our results show that the Ska complex interacts with the structured regions of tubulin monomers, mainly at helices H3 and H4 of β-tubulin and H12 of α-tubulin, whose accessibility is not perturbed on MTs assuming different conformations. This feature gives the Ska complex the ability to bind both straight and curved protofilaments with equal efficiency (Fig. 7). One of the sites (Glu110, 156 and 162 in H3) through which β-tubulin makes contact with the Ska1–MTBD is particularly intriguing, as this site is close to the GTP-binding site and also near the regions involved in lateral contacts between adjacent MT protofilaments. Furthermore, this site is recognized by EB1 and has been suggested to be important for EB1’s end-tracking activity and for stabilizing growing MTs^48^. In this context, it would be interesting to know whether the Ska complex can also influence MT dynamics.

One of the intriguing observations made in our study concerns the ability of the Ska complex to bind MTs in multiple different orientations. This combines with the fact that the Ska1–MTBDs are loosely connected to the W-shaped triple helical structure through a 40 amino acid loop, thus providing additional flexibility that may be important to allow the KTs to track disassembling MTs (Fig. 7). This is in stark contrast to the Ndc80 complex, where CH domains of Ndc80 are connected to a rather rigid helical bundle^13^,^33^. We further envisage that the presence of this loop in Ska1 is likely to be critical for efficient MT tracking by the Ska complex and possibly for mediating protein–protein interactions with other KT components. In support of this view, we found that deletion of the Ska1 loop delays mitotic progression of dividing cells.

In summary, our results indicate that the function of the Ska complex is conferred by its ability to interact with regions of tubulin monomers whose accessibility is not affected by different MT structures. These interactions involve multipartite binding sites and allow MT binding in multiple orientations (Fig. 7). Future structural and functional studies on whether and how Ska–MT interactions are modulated by the presence of other MT-binding proteins, notably the Ndc80 complex, will advance our understanding of the molecular underpinnings of KT–MT attachments and chromosome segregation.

## Methods

### Expression and purification of recombinant proteins

Ska1_133–255_ was cloned into a pEC-K-3C-His-GST vector as an N-terminally His-GST-tagged protein with a 3C-cleavage site. Ska1, Ska2 and Ska3 V58I were cloned individually in a pEC-S-CDF-His, pEC-A-HT-His GST and pEC-K-HT-His vectors, respectively, with TEV cleavage sites. Ska1 mutants were generated following the Quikchange site-directed mutagenesis method (Stratagene; primer details are given in Supplementary Table S3). To express the Ska complex containing Ska1Δloop, Ska1Δloop(GSSG) and phosphomimic mutants, Quikchange site-directed mutagenesis was performed on a polycistronic vector (primer details are given in Supplementary Table S3), pETMCN (gift from C. Romier, IGBMC, Strasbourg) containing GST-Ska2 (3C-cleavable), untagged Ska1 and Ska3. All protein complexes were expressed in *Escherichia coli* strain BL21 Gold, either using the polycistronic constructs or by cotransforming all the three plasmids containing individual Ska components. Cultures were induced overnight at 18 °C and purified using a similar protocol. Cells were lysed in a buffer containing 20 mM Tris, pH 8, 500 mM NaCl and 5 mM dithiothreitol (DTT). The protein complexes were purified by affinity chromatography in batch mode using glutathione sepharose (GE Healthcare) beads. Protein-bound beads were washed with 20 mM Tris, pH 8, 500 mM NaCl and 5 mM DTT, followed by 20 mM Tris, pH 8, 1 M NaCl, 50 mM KCl, 10 mM MgCl_2_, 2 mM ATP and 5 mM DTT, then finally with 20 mM Tris, pH 8, 100 mM NaCl and 5 mM DTT. Proteins with 3C-cleavage sites were cleaved, while the proteins were still bound to the beads. TEV cleavable proteins were eluted with 50 mM glutathione, 20 mM Tris, pH 8, 100 mM NaCl and 5 mM DTT, and the tags were removed in solution overnight. Subsequently, proteins/protein complexes were purified by size-exclusion chromatography in 20 mM Tris, pH 8, 100 mM NaCl and 5 mM DTT (Superose 6, GE Healthcare). Ndc80 Bonsai (kindly gifted by Andrea Musacchio) was expressed in *E. coli* BL21 (DE3)^13^. Cells were lysed in lysis buffer containing 50 mM Tris, pH 7.6, 300 mM NaCl, 1 mM DTT and 1 mM EDTA. Cleared lysate was incubated with glutathione sepharose beads. After 3 h incubation at 4 °C, beads were washed with 50 mM Tris, pH 7.6, 150 mM NaCl, 1 mM DTT and 1 mM EDTA, and cleaved with 3C protease for 16 h at 4 °C. Concentrated protein was loaded onto a Superose 6 size-exclusion chromatography column (GE Healthcare) equilibrated with 20 mM Tris, pH 7.6, 100 mM NaCl, 1 mM DTT and 10% glycerol.

### Crystallization and data collection

Crystallization trials were performed using a nanolitre crystallization robot at the Edinburgh Protein Production Facility. Crystals of form I (C222_1_) were grown by vapour diffusion method using Morpheus condition C2, mother liquor containing 0.1 M Imidazole-MES buffer, 0.09 M NPS mix (NaNO_3_, Na_2_HPO_4_, (NH_4_)_2_SO_4_) and 30% EDO_P8K (ethylene glycol; PEG 8 K) (with 1 μl of 15–20 mg ml^−1^ protein sample mixed with 1 μl of mother liquor). Crystals of form II (P3_2_) were grown in mother liquor containing 24% (w/v) PEG 1,500 and 20% glycerol. As the crystallization conditions were suitable to act as cryoprotectants, crystals were directly flash frozen in liquid nitrogen. The crystals diffracted to about 2 Å resolution at the MX beamlines of the Diamond Light Source (Table 1).

### Crystal structure solution and refinement

The structure of the Ska1–MTBD (form I) was determined by the SAD method, using the data collected at the selenium (SE) edge (0.97 Å). Data was processed using XDS and scaled with SCALA of CCP4 (ref. 49). SAD phasing and the calculation of the initial map were performed using phenix.autosol from the PHENIX suite of programmes^50^. The model was built by iterative rounds of manual building with COOT^51^ and refinement using phenix.refine of PHENIX suite of programmes^50^. The structure of the Ska1–MTBD from P3_2_ space group was determined using molecular replacement method using PHENIX suite of programmes^50^. Data collection, phasing and refinement statistics are shown in Table 1.

### Molecular dynamics simulations

For the MDS, chain B of crystal form I (C222_1_) and model 1 of NMR structure of the *C. elegans* (pdb: 2LYC) was used. MD studies were carried out using the AMBER12 (ref. 52) package. The missing atoms were built using standard geometries as implemented in AMBER. Each system was solvated with a box of TIP3P water molecules such that the boundary of the box was at least 10 Å from any protein atom. The net positive charges in the system were balanced by adding chloride ions. The force field ff12SB was used for intermolecular interactions. The particle mesh Ewald method was used for treating the long-range electrostatics. All bonds involving hydrogen were constrained by SHAKE. An integration time step of 2 fs was used for propagating the dynamics. Each system was initially minimized for 3,000 steps to remove any unfavourable interactions between the protein and the solvent, followed by heating to 300 K over 30 ps under normal pressure/temperature conditions. Subsequently, each system was simulated for 50 ns at constant temperature (300 K) and pressure (1 atm), and the structures were stored every 10 ps for analysis. Analysis was carried out using VMD^53^.

### MT cosedimentation assays

Tubulin was purchased from Cytoskeleton Inc. and MTs were polymerized according to manufacturer’s instructions. To generate vinblastine spirals, tubulin was diluted to 3 mg ml^−1^ in 80 mM PIPES, pH 6.8, 1 mM EGTA, 1 mM MgCl_2_, 1 mM DTT and 5% sucrose supplemented with 3 mM vinblastine sulphate (Sigma-Aldrich) at room temperature for 2 h. Subtilisin-treated MTs were obtained after incubation of 6 μM taxol-stabilized MTs for 45 min at 30 °C with 100 μg ml^−1^ subtilisin A (Sigma-Aldrich) following a previously reported procedure^13^. The reaction was stopped with 10 mM phenylmethyl sulphonyl fluoride and the digested MTs pelleted (434,400*g*, TLA 100.3, 10 min, 25 °C) and resuspended in the original volume of general tubulin buffer (80 mM PIPES, pH 6.9, 2 mM MgCl_2_ and 0.5 mM EGTA).

For MT-pelleting assays, taxol-stabilized MTs, vinblastine spirals or subtilisin-treated MTs (0–12 μM tubulin dimer as stated in each experiment) were incubated at room temperature for 10 min with 1 or 3 μM protein (Ska or Ndc80 bonsai) in a 50-μl reaction volume in BRB80 buffer (80 mM PIPES, pH 6.9, 1 mM EGTA and 1 mM MgCl_2_) with 100 mM NaCl and 4 mM DTT in the presence of 20 μM taxol or 3 mM vinblastine. The reaction was then layered onto a 250-μl glycerol cushion buffer (BRB80, 50% glycerol, 4 mM DTT for taxol-stabilized MTs and BRB80, 30% glycerol, 4 mM DTT for vinblastine spirals with the appropriate drug) and ultracentrifuged for 10 min at 434,400*g* in a Beckman TLA 100.3 rotor at 25 °C. Pellets and supernatants were analysed by SDS–PAGE. Gels were stained with Coomassie Blue gel staining and quantification was performed with ImageJ^54^. Normalized binding data were obtained by dividing the values of the pellet fraction by the sum of pellet and supernatant. Fitting analysis and *K*_d_ calculations were carried out using GraphPad Prism, version 6.0 (GraphPad Software, Inc).

For the Aurora B assay, we incubated 3 μM Ska complex or Ndc80 bonsai with 10 mM ATP, 20 mM MgSO_4_ and 900 nM Aurora B for 30 min at 30 °C. Taxol-stabilized MTs (6 μM tubulin dimer) were then added for 10 min at room temperature and cosedimentation assays were performed as described above. In the second batch of experiments, we first incubated the protein (Ska complex or Ndc80 bonsai) with the taxol-stabilized MTs for 10 min and then after we incubated for 30 min at 30 °C with Aurora B.

### Chemical crosslinking and MS analysis

Crosslinking experiments were carried out using a zero-length crosslinking agent, EDC (Thermo Fisher Scientific) in the presence of *N*-hydroxysulphosuccinimide (Thermo Fisher Scientific). Ska complex (6 μM) and 10 μM MTs were incubated with 10 μg EDC and 22 μg *N*-hydroxysulphosuccinimide in a final volume of 20 μl. The reaction mixture was incubated for 90 min at 25 °C and was quenched by adding Tris-Cl to a final concentration of 100 mM. The reactions were resolved by SDS–PAGE (4–12% Bis-Tris NuPAGE, Invitrogen) gel separation and stained using Instant Blue (Expedeon). The bands corresponding to crosslinked complexes were excised and the proteins therein were reduced using 10 mM DTT for 30 min at room temperature, alkylated with 55 mM iodoacetamide for 20 min in the dark at room temperature and digested using 13 ng μl^−1^ trypsin (sequencing grade; Promega) overnight at 37 °C (ref. 14). The digested peptides were desalted using C18-Stage-Tips^55^ and analysed on a LTQ Orbitrap Velos mass spectrometer (Thermo Fisher Scientific)^56^,^57^,^58^. An analytical column with a spray emitter (75 μm inner diameter, 8 μm opening, 250 mM length; New Objectives) that was packed with C18 material (ReproSil-Pur C18-AQ 3 μm; Dr Maisch GmbH, Ammerbuch-Entringen, Germany) using an air pressure pump (Proxeon Biosystems). Mobile phase A consisted of water with 0.1% formic acid. Mobile phase B consisted of 80% acetonitrile with 0.1% formic acid. Peptides were loaded onto the column with 1% B at 600 nl min^−1^ flow rate and eluted at 300 nl min^−1^ flow rate, with a linear gradient increased from 5 to 35% acetonitrile in 0.1% formic acid in 150 min to elute peptides. Peptides were analysed using a high/high strategy; both MS spectra and MS2 spectra were acquired in the Orbitrap^56^. Mass spectra were recorded at 100,000 resolution. The eight highest intensity peaks with a charge state of three or higher were selected in each cycle for ion-trap fragmentation. The fragments were produced using collision-induced dissociation with 35% normalized collision energy and detected by the Orbitrap at 7,500 resolution. Dynamic exclusion was set to 30 s and repeat count was 1. The data were processed, generating peak lists by MaxQuant^59^ and matching crosslinked peptides to spectra using in-house developed Xi software.

### Cell culture and siRNA depletion

HeLa S3, and HeLa S3 cells expressing histone H2B-GFP^60^, were routinely maintained in DMEM (Invitrogen) supplemented with 10% fetal bovine serum and penicillin/streptomycin (100 IU ml^−1^ and 100 mg ml^−1^, respectively; Gibco). For synchronization studies, cells were arrested for 20 h with 2 mM thymidine, followed by a release into fresh medium for 6–8 h and a second thymidine block of 16 h and release for 10 h before fixation or visualization^30^,^36^. All Ska constructs were generated in the pcDNA3.1 plasmid (Invitrogen), driven by the cytomegalovirus promoter, and modified to carry an N-terminal triple-Myc tag or a single mCherry tag. For rescue experiments, Ska1 siRNA-resistant constructs were used. Plasmid transfections were performed using TransIT-LT1 reagent (Mirus Bio Corporation) according to the manufacturer’s instructions. siRNA duplexes were transfected using Oligofectamine (Invitrogen) according to the manufacturer’s instructions. The sequences of the Ska1 (ref. 27) and control GL2 duplexes^61^ are: 5′-CCCGCTTAACCTATAATCAAA-3′ and 5′-AACGTACGCGGAATACTTCGA-3′, respectively. For western blotting, a rabbit anti-Ska1 antibody (1:1,000) and a mouse anti-α-Tubulin antibody (DM1α 1:2,000; Sigma) were used^27^.

### Immunofluorescence and time-lapse microscopy

Cells grown on coverslips were fixed and permeabilized simultaneously in PTEMF buffer (0.2% Triton X-100, 20 mM PIPES, pH 6.8, 1 mM MgCl2, 10 mM EGTA and 4% formaldehyde). Cells were stained with mouse anti-myc 9E10 monoclonal antibody (culture supernatant, 1:2) and human CREST autoimmune serum (1:2,000; Immunovision). DNA was visualized with 4′,6-diamidino-2-phenylindole (2 μg ml^−1^). All primary antibodies were detected with Cy2/Cy3-conjugated donkey antibodies (Dianova). A Deltavision microscope (Applied Precision) was used for immunofluorescence processing and image acquisition^60^. For time-lapse microscopy, all treatments within a single experiment were performed simultaneously. Cells were imaged using a Nikon ECLIPSE Ti microscope equipped with a CoolLED pE-1 excitation system and a 20 × /0.75 air Plan Apo objective (Nikon). During imaging, the atmosphere was maintained at a temperature of 37 °C, humidity 60 and 5% CO_2_. Images were captured at 5-min intervals for 22 h at multiple positions. Green fluorescent protein and mCherry fluorescence images were acquired at each time point with 30 ms and 60 ms exposure times, respectively. mCherry fluorescence was imaged only every five time point to monitor transfected cells. MetaMorph 7.7 software (MDS Analytical Technologies) was used to collect and process data.

## Additional information

**How to cite this article:** Abad, M. A. *et al*. Structural basis for microtubule recognition by the human kinetochore Ska complex. *Nat. Commun.* 5:2964 doi: 10.1038/ncomms3964 (2014).

**Accession codes:** Coordinates and structure factors have been deposited in the protein data bank under accession codes 4C9Y (C222_1_ form) and 4CA0 (P3_2_ form).

## Supplementary Material

Supplementary Figures and Tables Supplementary Figures S1-S9 and Supplementary Tables S1-S3

Supplementary Movie 1 Time-lapse imaging of HeLa S3 cells stably expressing H2B-GFP treated with GL2 siRNA and transfected with an empty mCherry plasmid. Imaging was performed in all cases using a microscope (Eclipse Ti) with a 20X objective for 22 h. Cell reflecting the phenotype shown by the majority of Ska1-depleted cells, with a metaphase-like appearance.

Supplementary Movie 2 Time-lapse imaging of HeLa S3 cells stably expressing H2B-GFP

Supplementary Movie 3 Time-lapse imaging of HeLa S3 cells stably expressing H2B-GFP treated with GL2 siRNA and transfected with mCherry-Ska1 WT cells progressed normally through mitosis.

Supplementary Movie 4 Time-lapse imaging of HeLa S3 cells stably expressing H2B-GFP treated with GL2 siRNA and transfected with mCherry-Ska1^1-132^. Anaphase onset was delayed and the frequency of apoptosis increased, but most cells showed aligned chromosomes.

Supplementary Movie 5 Time-lapse imaging of HeLa S3 cells stably expressing H2B-GFP treated with GL2 siRNA and transfected with mCherry-Ska1-MTBD. Cells exhibit delayed chromosome alignment and problems in maintaining a tight metaphase plate.

Supplementary Movie 6 Time-lapse imaging of HeLa S3 cells stably expressing H2B-GFP treated with GL2 siRNA and transfected with mCherry- Ska1δloop(GSSG). Cells delayed both initial chromosome alignment and anaphase onset by two fold.

Supplementary Movie 7 Time-lapse imaging of HeLa S3 cells stably expressing H2B-GFP treated with GL2 siRNA and transfected with mCherry-Ska1 R155/236/245A. Cells showed perturbed mitotic progression, characterized by a prolonged delay in anaphase onset.

Supplementary Movie 8 Time-lapse imaging of HeLa S3 cells stably expressing H2B-GFP treated with GL2 siRNA and transfected with mCherry- Ska1 K217/223/226/227A. Cells showed perturbed mitotic progression, characterized by a prolonged delay in anaphase onset.

Supplementary Movie 9 Time-lapse imaging of HeLa S3 cells stably expressing H2B-GFP treated with GL2 siRNA and transfected with mCherry- Ska1 K183/184/203/206A. Cells showed perturbed mitotic progression, characterized by a prolonged delay in anaphase onset.

Supplementary Movie 10 Normal mode analysis of MD-simulated structures of human Ska1-MTBD.

## Figures and Tables

**Table 1:** Data collection, phasing and refinement statistics.

	**Crystal form 1**	**Crystal form 2**
*Data collection*		
Space group	C222_1_	P3_2_
*Cell dimensions*		
*a*, *b*, *c* (Å)	39.01, 161.58, 104.48	47.18, 47.18, 116.50
*α*, *β*, *γ* (°)	90, 90, 90	90, 90, 120
	Peak	
Wavelength	0.98	1.541
Resolution (Å)	63.9–2.0 (2.12–2.01)	58.3–2.3 (2.37–2.25)
*R*_merge_	7.8 (45.5)	4.8 (30.8)
*I*/σ*I*	15.2 (3.8)	17.3 (3.6)
Completeness (%)	99.8 (99.6)	98.5 (90.1)
Redundancy	7.5 (7.5)	4.5 (4.0)
		
*Refinement*		
Resolution (Å)	27.0–2.1	58.3–2.3
No. of reflections	169,025	60,423
*R*_work_/*R*_free_	19.4/24.6	22.5/26.8
No. of atoms	2,161	
Protein	2,026	1,962
Water	135	40
*β-Factors*		
Protein	52.9	68.2
Water	50.5	50.3
*Root mean square deviation*		
Bond lengths (Å)	0.008	0.008
Bond angles (°)	1.09	1.12

Values in parentheses are for highest-resolution shell.
