# Supplementary material for: Structural basis for microtubule recognition by the human kinetochore Ska complex
Source: Nat Commun. 2014 Jan 13;5:2964. doi: 10.1038/ncomms3964 (PMC3923297; doi:10.1038/ncomms3964)
Supplement: Supplementary Figures and Tables — Supplementary Figures S1-S9 and Supplementary Tables S1-S3 [file ncomms3964-s1.pdf]

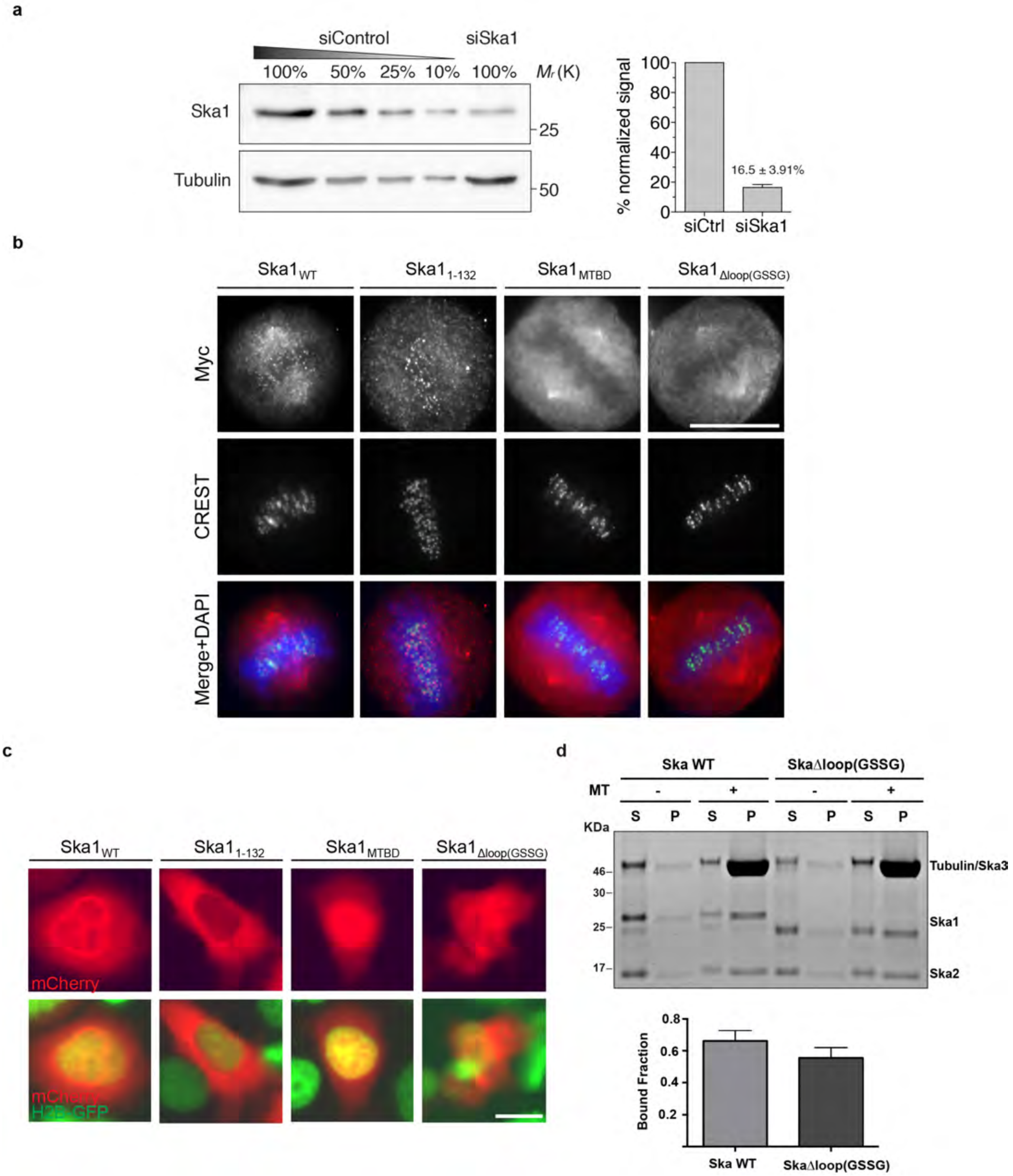

Supplementary Figure S1: The Ska1-loop is required for the correct localization of the Ska complex. (**a**) Cells were treated with 45nM of the indicated siRNAs, synchronized by a double thymidine arrest and transfected with a control plasmid as in **Fig. 1c** and **1d**. Left, efficiency of depletion assessed by Western blot. Right, quantification of the Ska1 signal intensity by densitometry (n = 4). (**b**) HeLa S3 cells were transfected with the indicated Myc -tagged siRNA-resistant constructs as previously before cells were fixed and stained with mouse anti-Myc (red), CREST serum (green) and DAPI. Bar, 10 µm. (**c**) Representative stills from time-lapse video-microscopy experiments illustrating the interphase localization of HeLa S3 cells stably expressing histone H2B-GFP and transfected with the indicated mCherry-tagged siRNA-resistant constructs as previously. Bar, 10 µm. (**d**) Top, representative coomassie blue staining of MT co-sedimentation assays as in **Fig. 1b** to check the influence of the loop region (92-132) on MT recognition together with the graph (bottom) corresponding to the quantification of the results obtained (mean±SD, n=3, *t*-test). Uncropped scans of the gels are shown in **Supplementary Fig. S9a and S9b**.

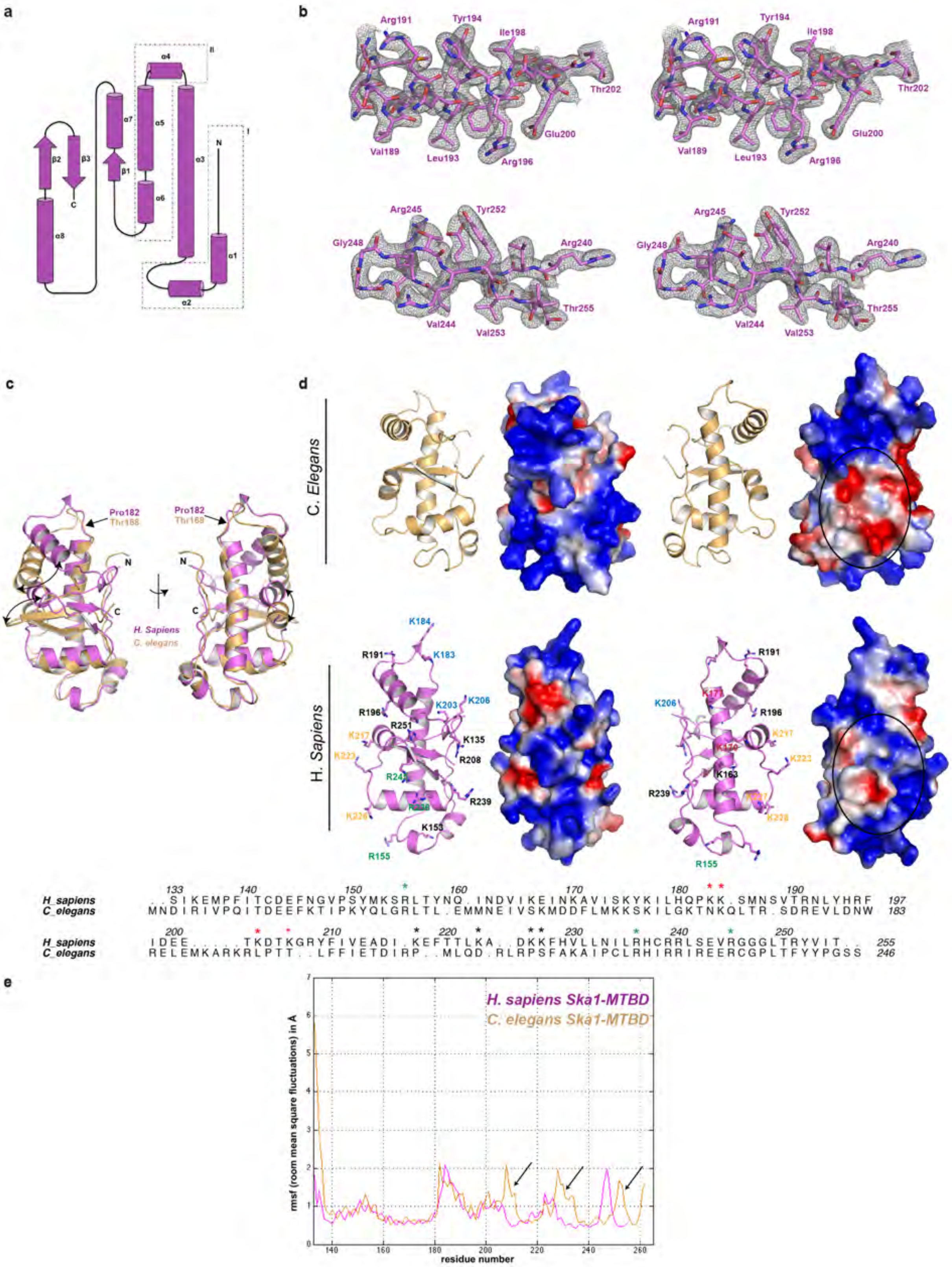

Supplementary Figure S2: Human and *C. elegans* Ska1-MTBDs exhibit distinctive surface properties due to differences in their intrinsic structural flexibility. (**a**) Topology diagram of human Ska1-MTBD. Secondary structure elements that are unique to Ska1 are highlighted in dotted lines as module I and II. (**b**) Electron density map (2fo-fc, contoured at 1σ) corresponding to regions helix α5 and beta strands β2 and β3 (regions where the crystal structure is different from NMR structure). (**c**) Structural superposition of human (magenta) and *C.elegans* (yellow; NMR structure, pdb: 2LYC) Ska1-MTBD. The residue at which conformational variation starts is highlighted by arrows (Pro182 in human and Thr168 in *C.elegans*). (**d**) Cartoon and surface representations of both human (bottom) and *C.elegans* (top) Ska1-MTBD revealing the differences in surface charge distribution. The region where surface charge distribution is strikingly different is highlighted. Pairwise sequence alignment of human and *C. elegans* Ska1-MTBD with residues critical for MT-binding highlighted with colour coded asterisks in consistent with **Fig. 2**. (**e**) Root mean square fluctuations of amino acid residues calculated from the trajectories obtained from 50ns molecular dynamic simulation. *C. elegans* residues that exhibit higher flexibility compared to their human counterpart are highlighted by arrows.

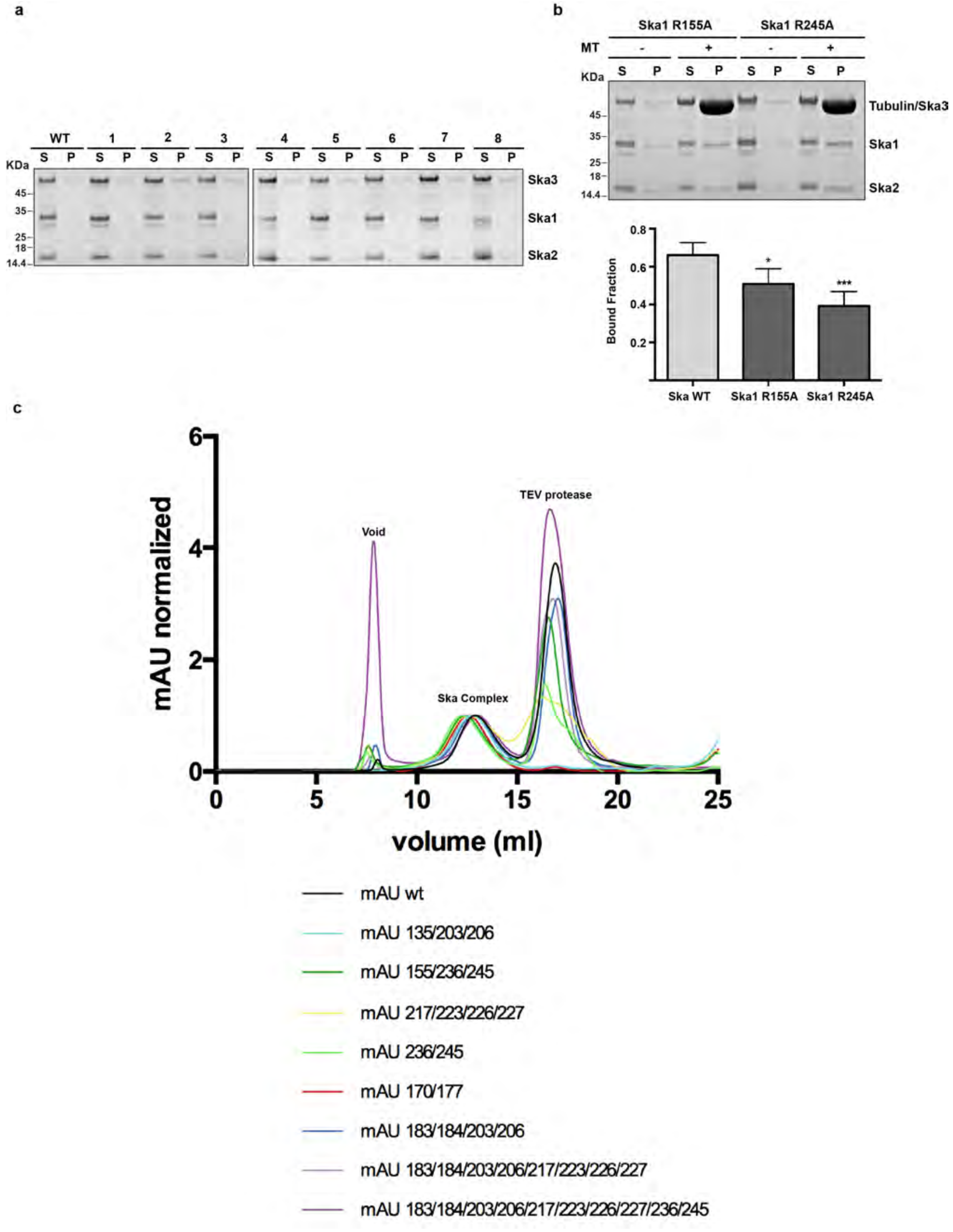

Supplementary Figure S3: Negative controls of mutants tested in Fig. 2 and evaluation of R155 and R245's contribution for MT-binding. (**a**) Representative SDS-PAGE of co-sedimentation assays with alanine mutants in identical conditions to the experiments in **Fig. 2B**, except in the absence of MTs. None of the mutant Ska complexes showed significant spurious pelleting. (**b**) Top, representative SDS-PAGE of MT-binding assay with the single alanine mutants R155A and R245A. Bottom, quantification of the results obtained (mean±SD, n=3, * $p \leq 0.05$, *** $p \leq 0.001$ *t*-test). Uncropped scans of the gels are shown in **Supplementary Fig. S9c** and **S9d**. (**c**) Comparison of the size exclusion chromatograms of all the mutants analysed in this study colour coded accordingly. Analysis shows that all mutants behave identically to the WT Ska complex.

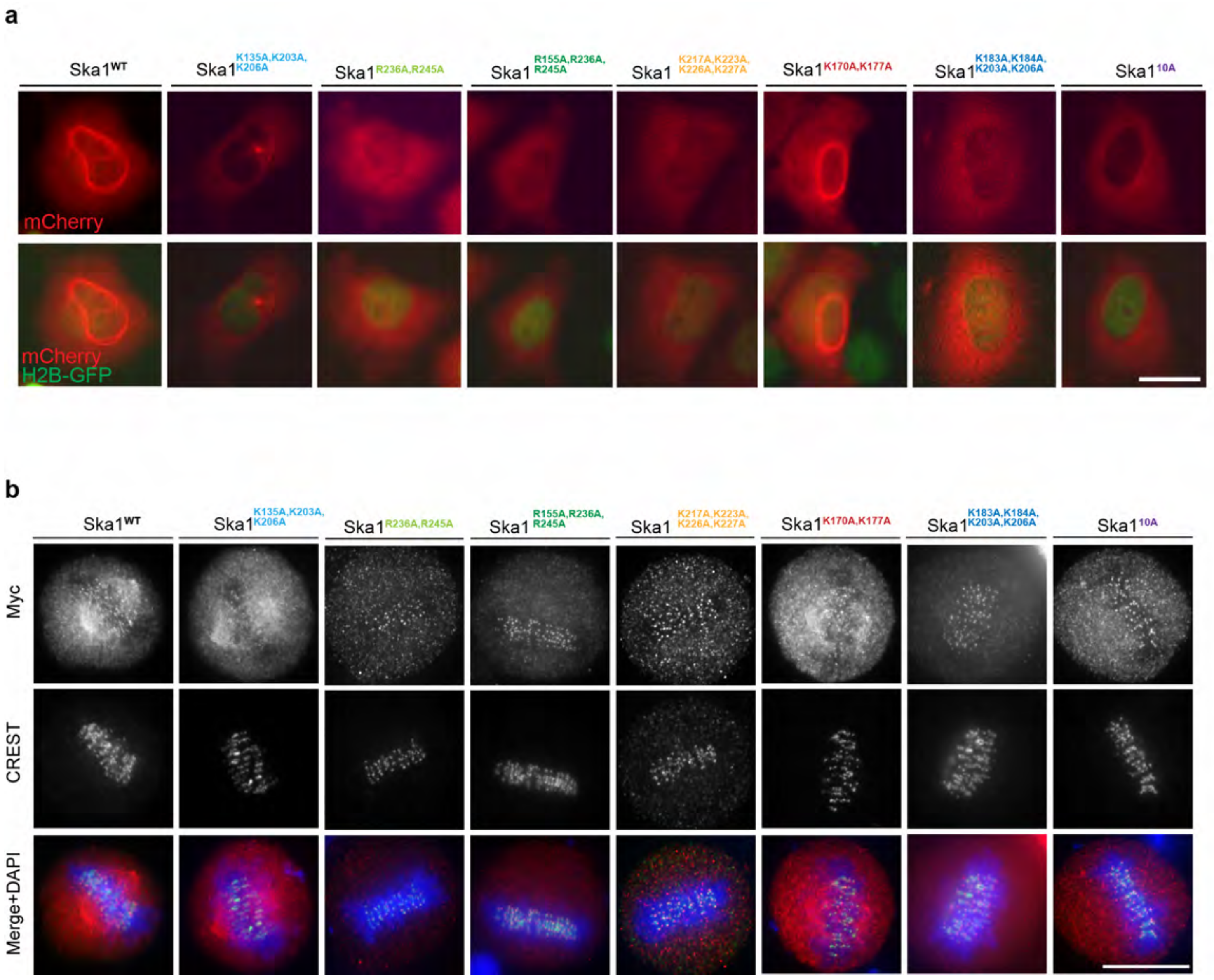

Supplementary Figure S4: *In vivo* analysis of the localization and MT-bundling activity of the different mutant Ska complexes. (**a**) Representative stills from time-lapse video-microscopy experiments illustrating the bundling ability *in vivo* of HeLa S3 cells stably expressing histone H2B-GFP and transfected with the indicated mCherry-tagged siRNA-resistant constructs as previously[30,36]. Bar, 10 µm. (**b**) HeLa S3 cells were transfected with the indicated Myc-tagged siRNA-resistant constructs as previously[30,36] before cells were fixed and stained with mouse anti-Myc (red), CREST serum (green) and DAPI. Bar, 10 µm.

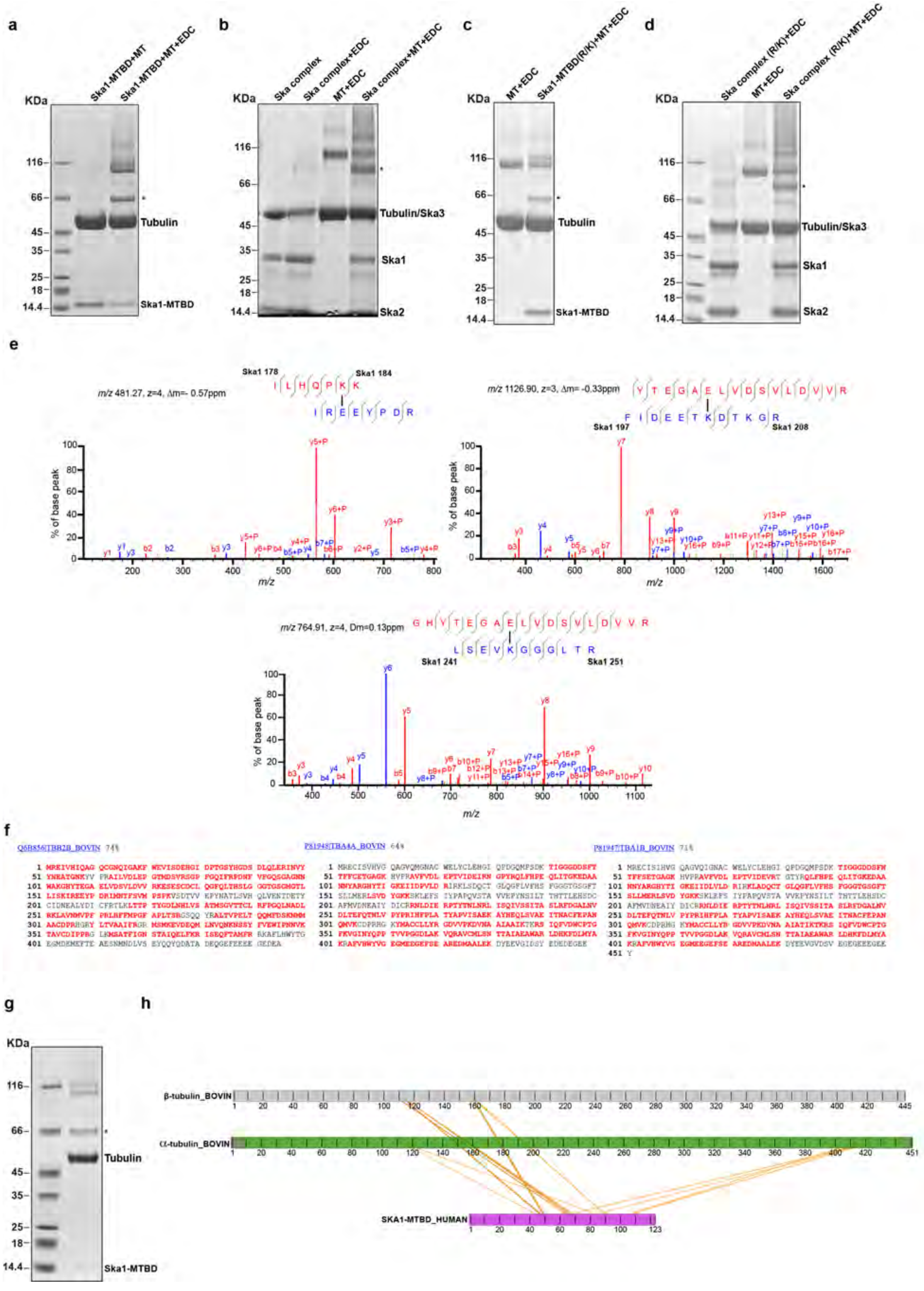

Supplementary Figure S5: Cross-linking/mass spectrometric analysis of Ska-MT interactions. Representative SDS-PAGEs corresponding to (**a**) Ska1-MTBD (**b**) Ska complex (**c**) Ska1-MTBD (R155/236/245K) and (**d**) Ska complex (R155/236/245K) incubated with EDC cross-linker and MTs. Bands analysed are marked with asterisks. (**e**) High resolution fragmentation spectra of a few representative cross-linked peptides. (**f**) Percentage sequence coverage of different isoforms of α and β tubulin with coverage highlighted in red. (**g**) Representative SDS-PAGE corresponding to Ska1-MTBD cross-linked to MTs and pelleted prior to analysis via MS. Uncropped scans of the gels are shown in **Supplementary Fig. S9e**. (**h**) Linkage map showing the sequence position of all the cross-linked residue pairs between Ska1-MTBD and MTs of pelleted samples.

a

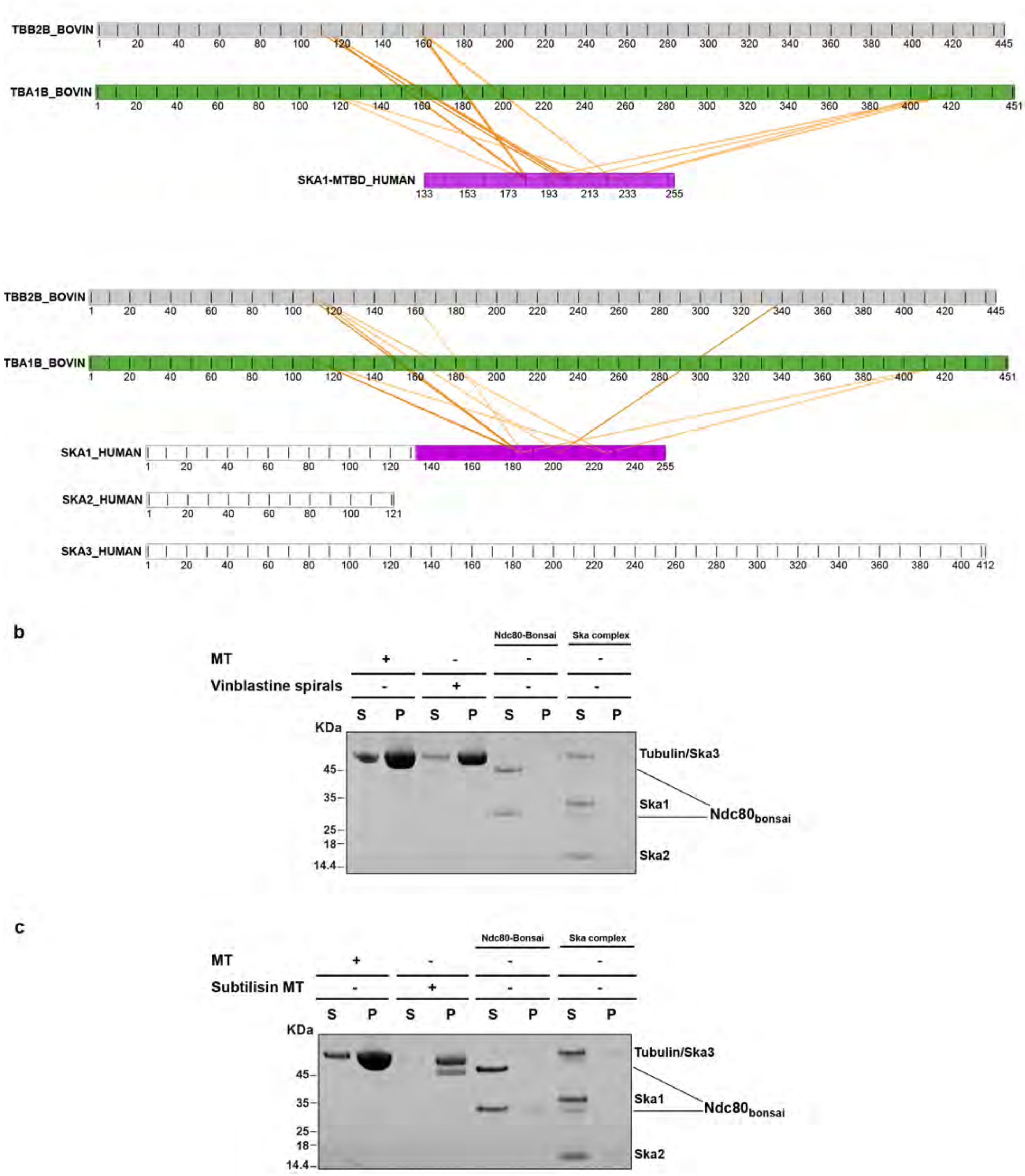

Supplementary Figure S6: The Ska complex binds straight and curved MTs with no apparent preference. (**a**) Linkage map showing the sequence position of all the cross-linked residue pairs between the Ska1-MTBD/Ska complex and Vinblastine spirals. (**b** and **c**) Representative SDS-PAGE of MT co-sedimentation assays showing proper (**b**) polymerization of MT and Vinblastine Spirals and (**c**) MT acidic tail digestion with subtilisin. Ndc80 bonsai and Ska complexes show no self-pelleting in the assays without MTs. Uncropped scans of the gels are shown in **Supplementary Fig. S9f**.

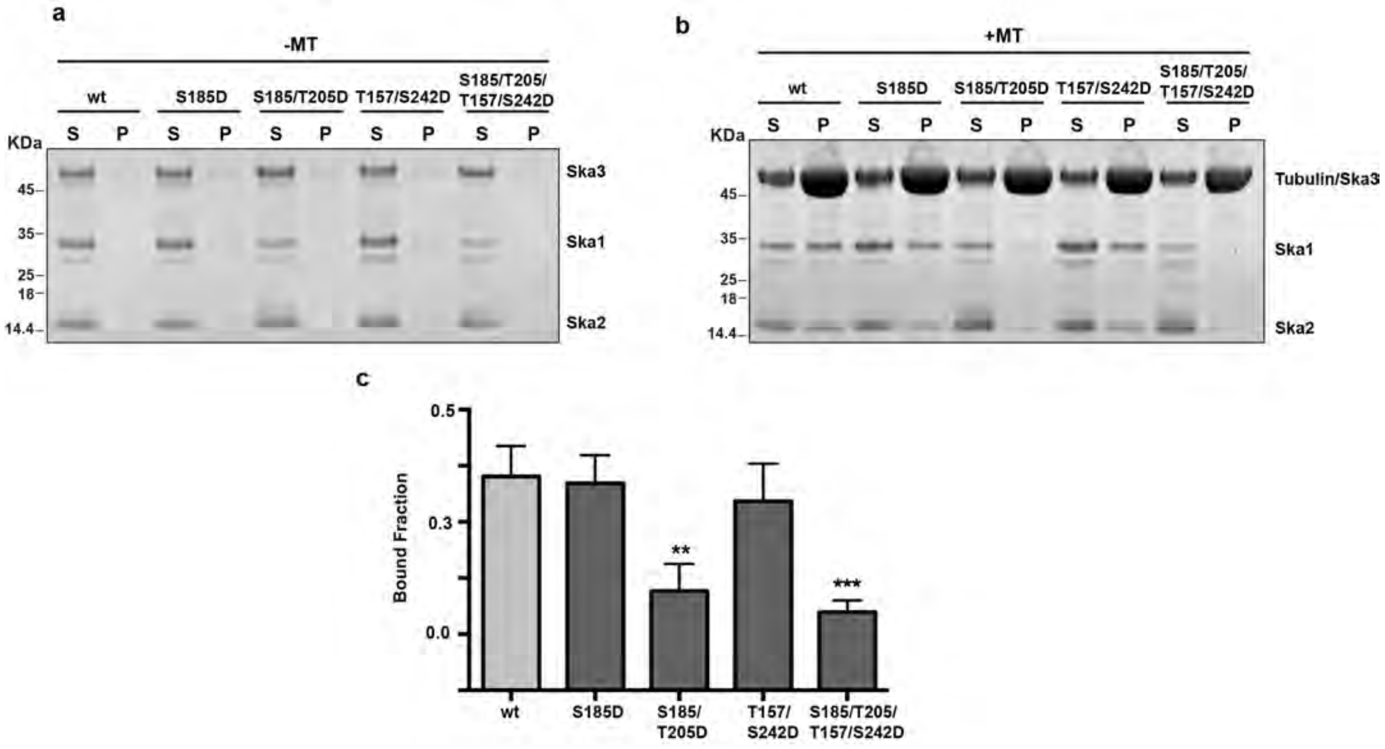

Supplementary Figure S7: Evaluation of the effect of constitutive phosphorylation of consensus Aurora B sites of Ska1, T157 and T242, on MT-binding. (**a**) Representative SDS-PAGE of MT co-sedimentation assay with the phospho-mimic mutant T157/242D. (**b**) Phospho-mimicking mutants evaluated in this study do not precipitate on its own during co-sedimentation assays. Representative SDS-PAGE of negative controls is shown here. Uncropped images of the gels are shown in **Supplementary Fig. S9g**. (**c**) Quantification for the binding assay shown in **a** (mean±SD, n=5, ** p≤0.01, *** p≤0.001 *t*-test).

a

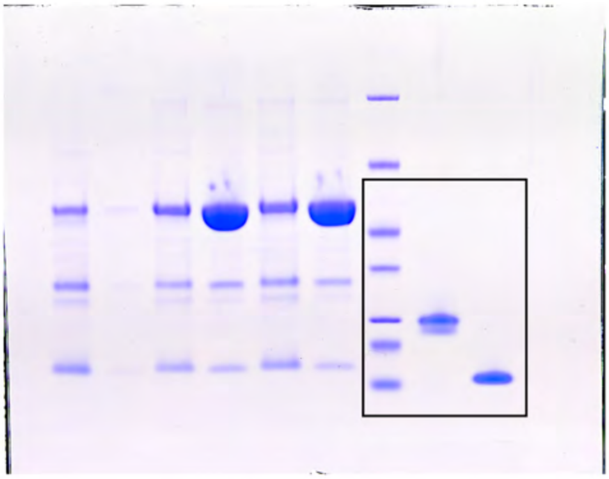

b

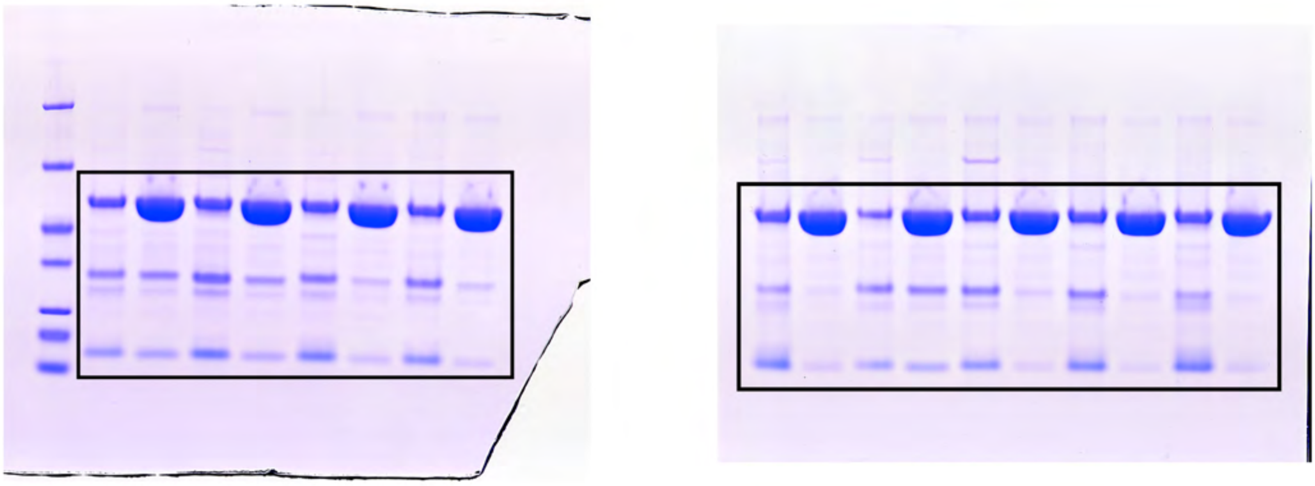

c

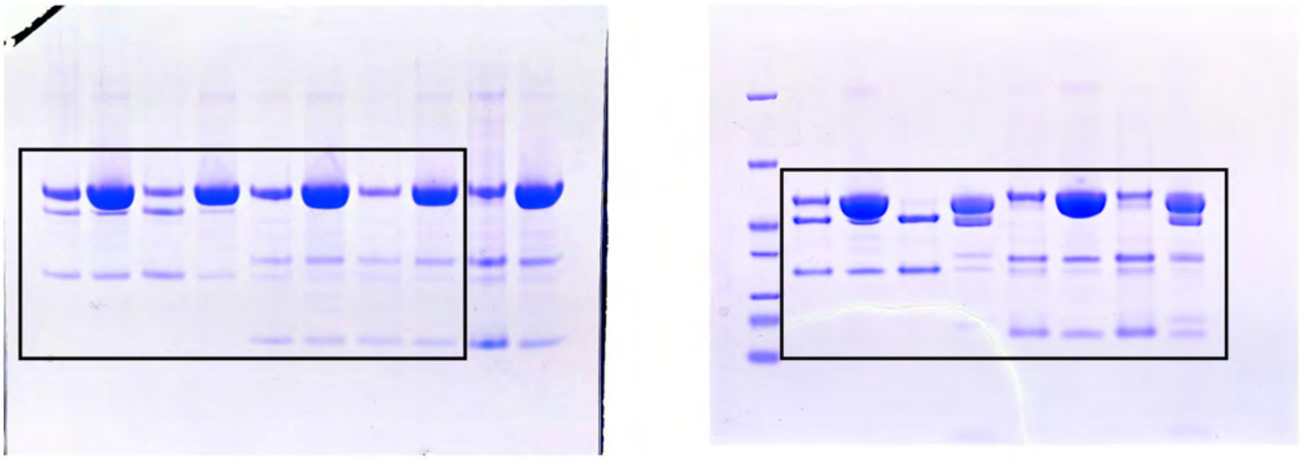

d

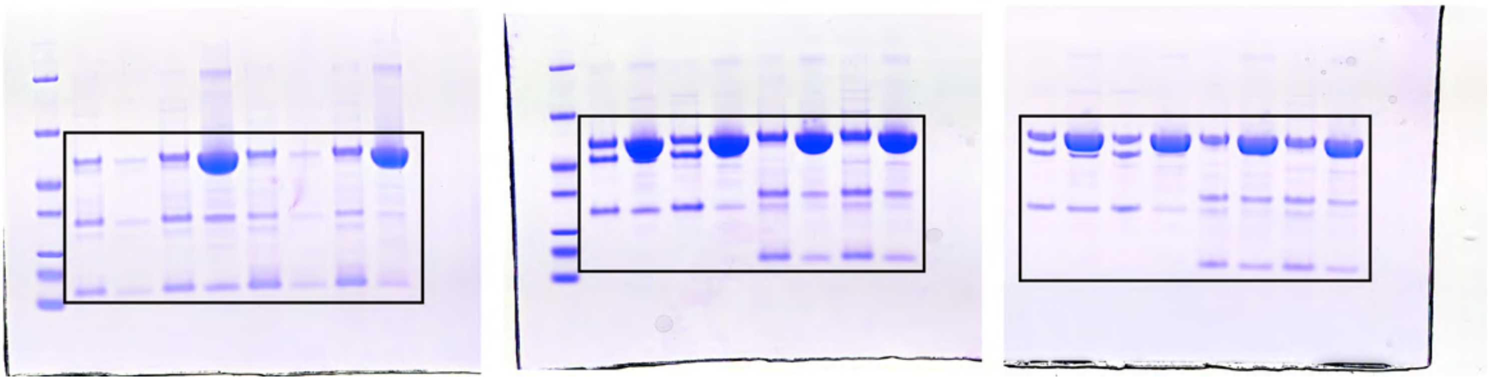

Supplementary Figure S8: Full scans of the gels shown in (a) Fig. 1a, (b) Fig. 2b, (c) Fig. 5a and 5c and (d) Fig. 6b, 6c and 6d.

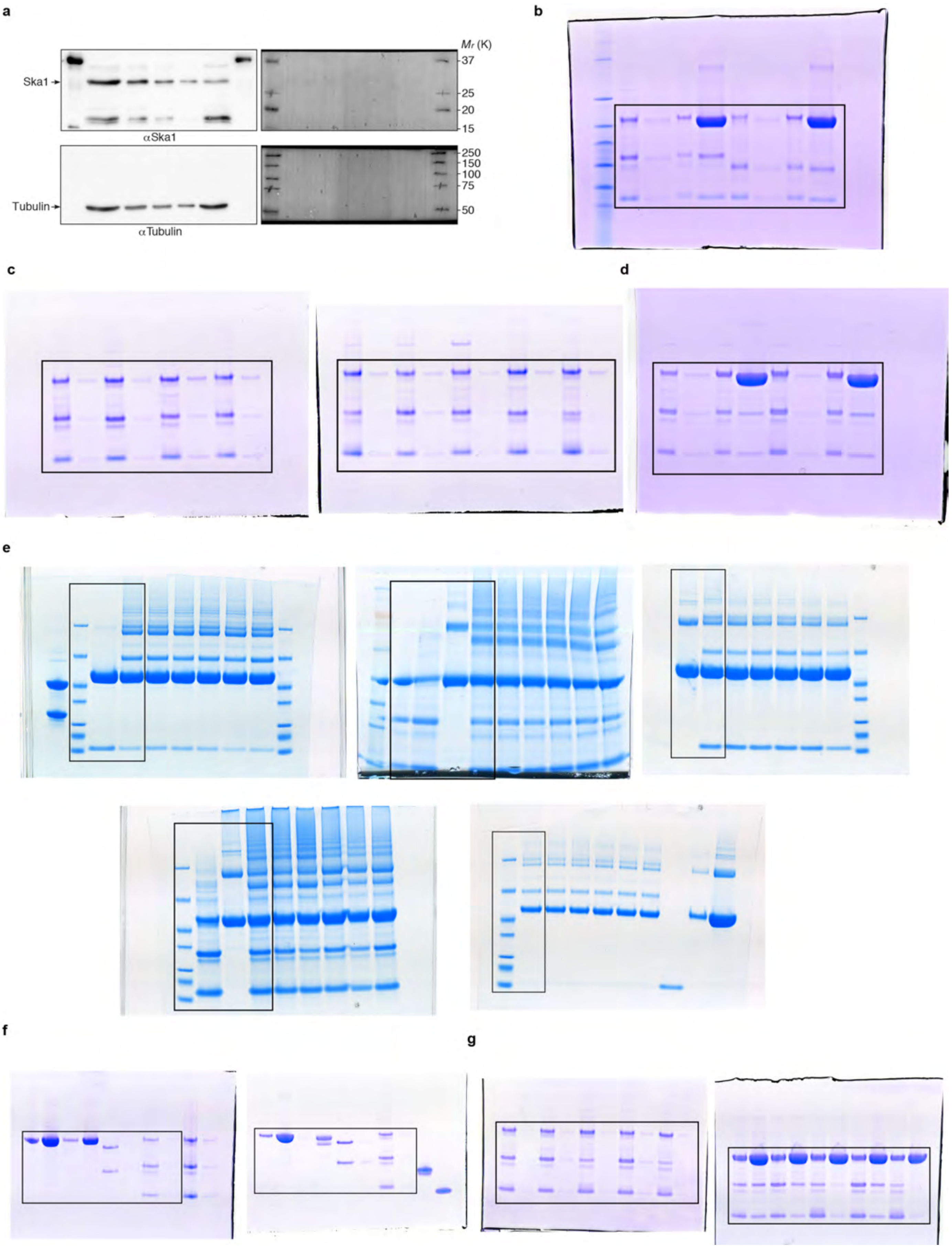

Supplementary Figure S9: Full scans of the gels shown in (a) Supplementary Fig. S1a, (b) Supplementary Fig. S1d, (c) Supplementary Fig. S3a, (d) Supplementary Fig. S3b, (e) Supplementary Fig. S5a, S5b, S5c, S5d and S5g, (f) Supplementary Fig. S6b and S6c and (g) Supplementary Fig S7a and S7b.

Supplementary Table S1. Mitotic timing of mutants shown in Fig. 1c and d

	NEBD-alignment	alignment-anaphase onset/death	NEBD-anaphase onset /death
**GL2 siRNA_V**	27	25	52
**Ska1 siRNA_V**	50	159	209
**Ska1 siRNA_WT**	20	34	54
**Ska1 siRNA_1-132**	25	171	196
**Ska1 siRNA MTBD**	57	113	170
**Ska1 siRNA_DLoop(GSSG)**	46	78	124

Average time in min. N>10 cells.

Supplementary Table S2. Mitotic timing of mutants shown in Fig. 3a and b

	**NEBD-alignment**	**alignment-anaphase onset/death**	**NEBD-anaphase onset /death**
**GL2 siRNA_V**	26	21	47
**Ska1 siRNA_V**	49	119	168
**Ska1 siRNA_WT**	25	35	60
**Ska1 siRNA_K183,K184,K203,K206A**	31	90	121
**Ska1 siRNA_K155,K236,K245A**	30	104	134
**Ska1 siRNA_K217,K223,K226,K227A**	28	125	153
**Ska1 siRNA_10A**	29	102	131

Average time in min. N>10 cells.

Supplementary Table S3. List of primers used in this study.

	Fwd	Rev
**Ska1-MTBD**	CCAGGGGCCCGACTCGATGAGTATTAAGGAATGC	CAGACCGCCACCGACTGCTTAGGTTATAACATAAC
**Ska1-Δloop**	P-AGCGGGAGTATTAAGGAAATGCCATTTATAAC	P-GCTCCCAACGTTTTCTTTAAGATGTTC
**Ska1 wt**	CCAGGGAGCAGCCTCGATGGCCTCGTCAGAT	GCAAAGCACCGGCCTCGTTAGGTTATAACATAA
**Site directed mutagenesis**		
**K203/206A**	TTTATTGATGAAGAAACGGCCGATACCGCCGGTCGTTATTTTATAGTG	CACTATAAAATAACGACCGGCGGTATCGGCCGTTTCTTCATCAATAAA
**K135A**	TAAAGAAGCCTCCCAAAGAGCAAAGAAGTATTGCGGAAATGCCATTTATAA	TTATAAATGGCATTTCCGCAATACTTCTTTGCTCTTTGGGAGGCTTCTTTA
**R236A**	TTTCACGTGTTACTGAATATTTTAGCACACTGCCGGAGG	CCTCCGGCAGTGTGCTAAAATATTCAGTAACACGTGAAA
**R245A**	AGGCTATCAGAGGTCGCAGGGGGAGGACTTAC	GTAAGTCCTCCCCCTGCGACCTCTGATAGCCT
**R155A**	GGTGTTCCTTCGTACATGAAATCCGCCTTAACCTATAATCAAATTAATGAT	ATCATTAATTTGATTATAGGTTAAGGCGGATTTCATGTACGAAGGAACACC
**K183/184A**	GTAAATATAAAATCCTACATCAGCCAGCAGCGTCTATGAATTCTGTGACCAGAA	GATTTCTGGTCACAGAATTCATAGACGCTGCTGGCTGATGTAGGATTTTATATT
**K217/223/226/227A**	GCTGACGCCGCCTTTCACGTGTTACTGAATATT	GGCCAAAGTTGTGAACTCGGCTATGTCAGCTTCC
**R236K**	AGAAGTTTCACGTGTTACTGAATATTTTAAAACACTGCCGGAGGCT	AGCCTCCGGCAGTGTTTTAAAATATTCAGTAACACGTGAAACTTCT
**R245K**	GGAGGCTATCAGAGGTCAAAGGGGGAGGACTTACTC	GAGTAAGTCCTCCCCCTTTGACCTCTGATAGCCTCC
**R155K**	GGTGTTCCTTCGTACATGAAATCCAAGTTAACCTATAATCAAATTAATGATG	CATCATTAATTTGATTATAGGTTAACTTGGATTTCATGTACGAAGGAACACC
**S185D**	CATCAGCCAAAAAAGGATATGAATTCTGTGAC	GTCACAGAATTCATATCCTTTTTTGGCTGATG
**T205D**	GAAACGAAGGATGACAAAGGTCGTTATTTTATAG	CTATAAAATAACGACCTTTGTCATCCTTCGTTTC
**T157D**	GAAATCCCGCTTAGACTATAATCAAATTAATG	CATTAATTTGATTATAGTCTAAGCGGGATTTC
**S242D**	CTGCCGGAGGCTAGATGAGGTCCGAGGGGG	CCCCCTCGGACCTCATCTAGCCTCCGGCAG
