# Supplementary figures and images for: Structural basis for microtubule recognition by the human kinetochore Ska complex
Source: Nat Commun. 2014 Jan 13;5:2964. doi: 10.1038/ncomms3964 (PMC3923297; doi:10.1038/ncomms3964)

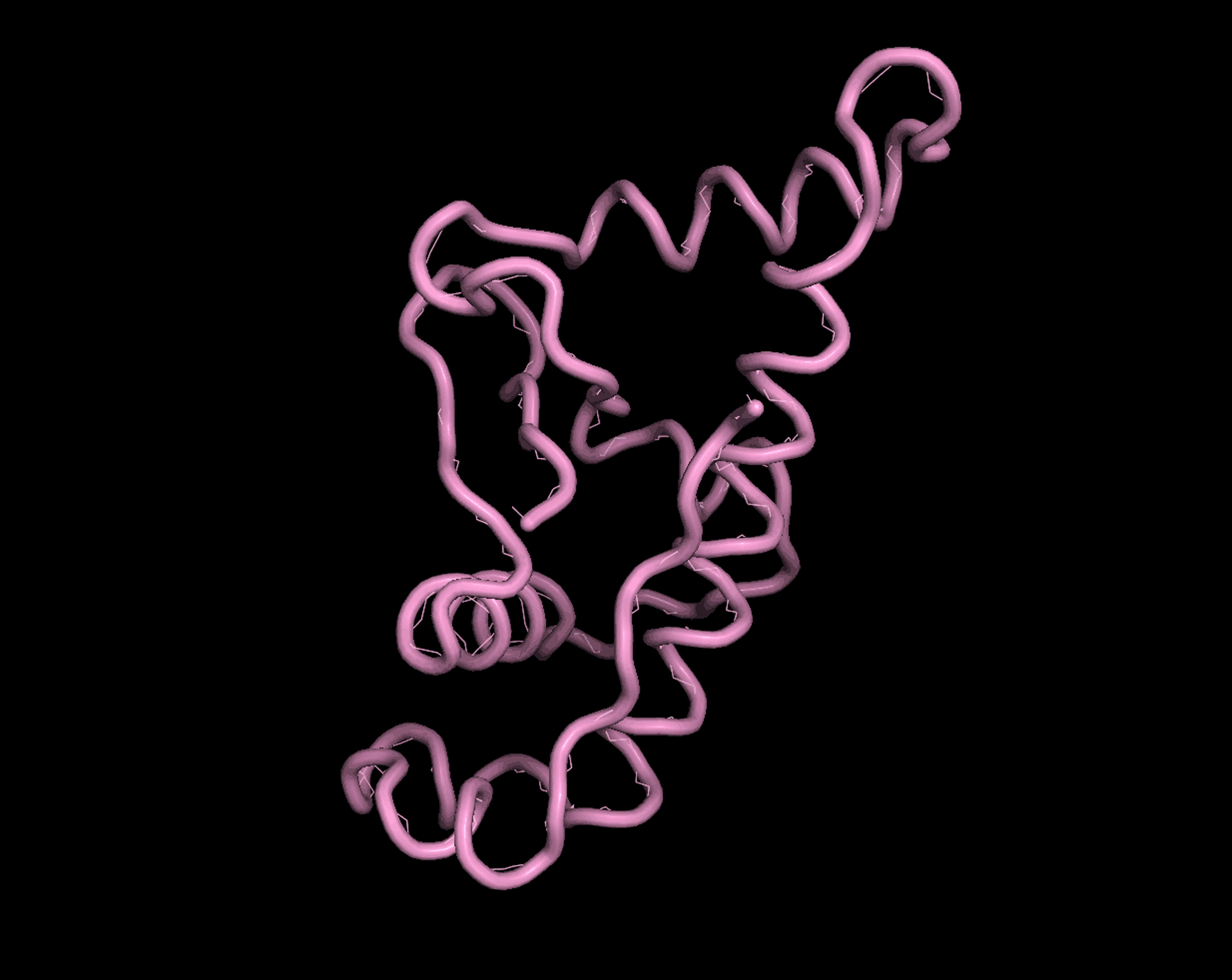

Supplement: Supplementary Movie 10 — Normal mode analysis of MD-simulated structures of human Ska1-MTBD. [file ncomms3964-s11.tif]
